# Supplementary material for: USP9X stabilizes XIAP to regulate mitotic cell death and chemoresistance in aggressive B‐cell lymphoma
Source: EMBO Mol Med. 2016 Jun 17;8(8):851–62. doi: 10.15252/emmm.201506047 (PMC4967940; doi:10.15252/emmm.201506047)
Supplement: Supplementary file 1 — Appendix [file EMMM-8-851-s001.pdf]

Appendix PDF

USP9X stabilizes XIAP to regulate mitotic cell death and chemoresistance in aggressive B-cell lymphoma

Katharina Engel, Martina Rudelius, Jolanta Slawska, Laura Jacobs, Behnaz Ahangarian Abhari, Bettina Altmann, Julia Kurutz, Abirami Rathakrishnan, Vanesa Fernandez-Sáiz, Andrä Brunner, Bianca-Sabrina Targosz, Felicia Loewecke, Christian Johannes Gloeckner, Marius Ueffing, Simone Fulda, Michael Pfreundschuh, Lorenz Trümper, Wolfram Klapper, Ulrich Keller, Philipp J. Jost, Andreas Rosenwald, Christian Peschel, and Florian Bassermann

Contents:

- **Appendix Figure S1 - USP9X protects XIAP from proteasomal degradation in mitosis**
- **Appendix Figure S2 - Silencing of *Usp9X* and *Xiap* reduces lymphoma infiltration and treatment resistance *in vivo***
- **Appendix Figure S3 - USP9X gene expression in ABC- and GCB-type DLBCL**
- **Appendix Table S1 - Patient characteristics**

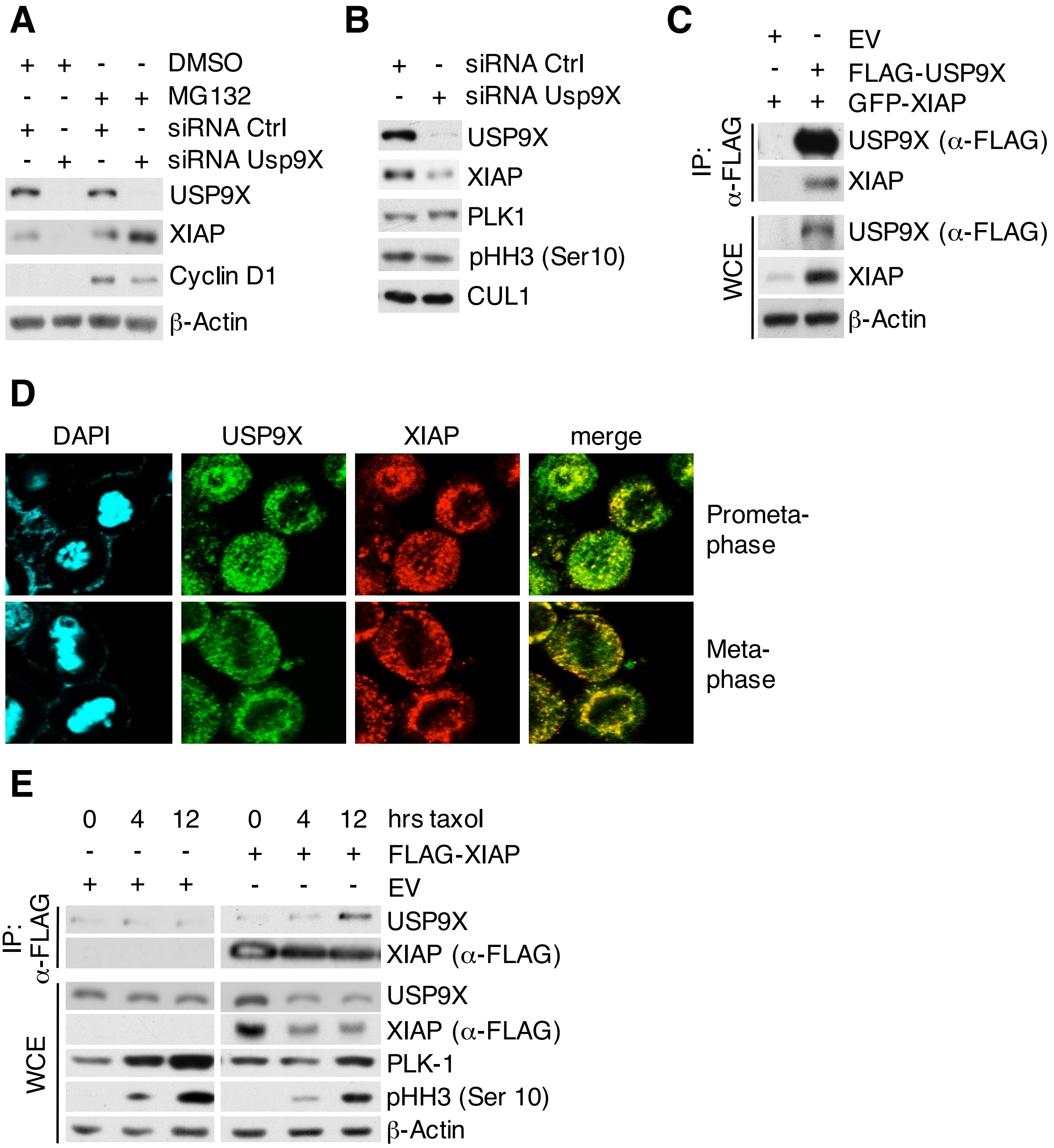

Appendix Figure S1 - USP9X protects XIAP from proteasomal degradation in mitosis

A Immunoblot analyses of HeLa cells that were arrested in mitosis by sequential thymidine-nocodazole exposure and treated with siRNAs against *USP9X* or a control transcript and DMSO or MG132 where indicated.

B Immunoblot analyses of mitotic HeLa cells, synchronized in G2 phase with the CDK1 inhibitor RO-3306 and then released for one hour to reach mitosis after knockdown of *USP9X* or a control transcript as indicated.

C Co-immunoprecipitation of USP9X and XIAP from mitotic HEK 293T cells that were transfected with GFP-tagged XIAP and FLAG-tagged USP9X or empty vector (EV) as indicated.

D Visualization of Cos7 cells by indirect immunofluorescence performed with antibodies to the indicated endogenous proteins. Prior to staining, cells were synchronized by sequential treatment with thymidine and nocodazole. Cells were then directly fixed to obtain prometaphase cells, or fixed 10 min after the release from the nocodazole block to obtain metaphase cells.

E Co-immunoprecipitation of XIAP with endogenous USP9X from HEK 293T cells that were transfected with FLAG-tagged XIAP or empty vector (EV) and treated with taxol as indicated.

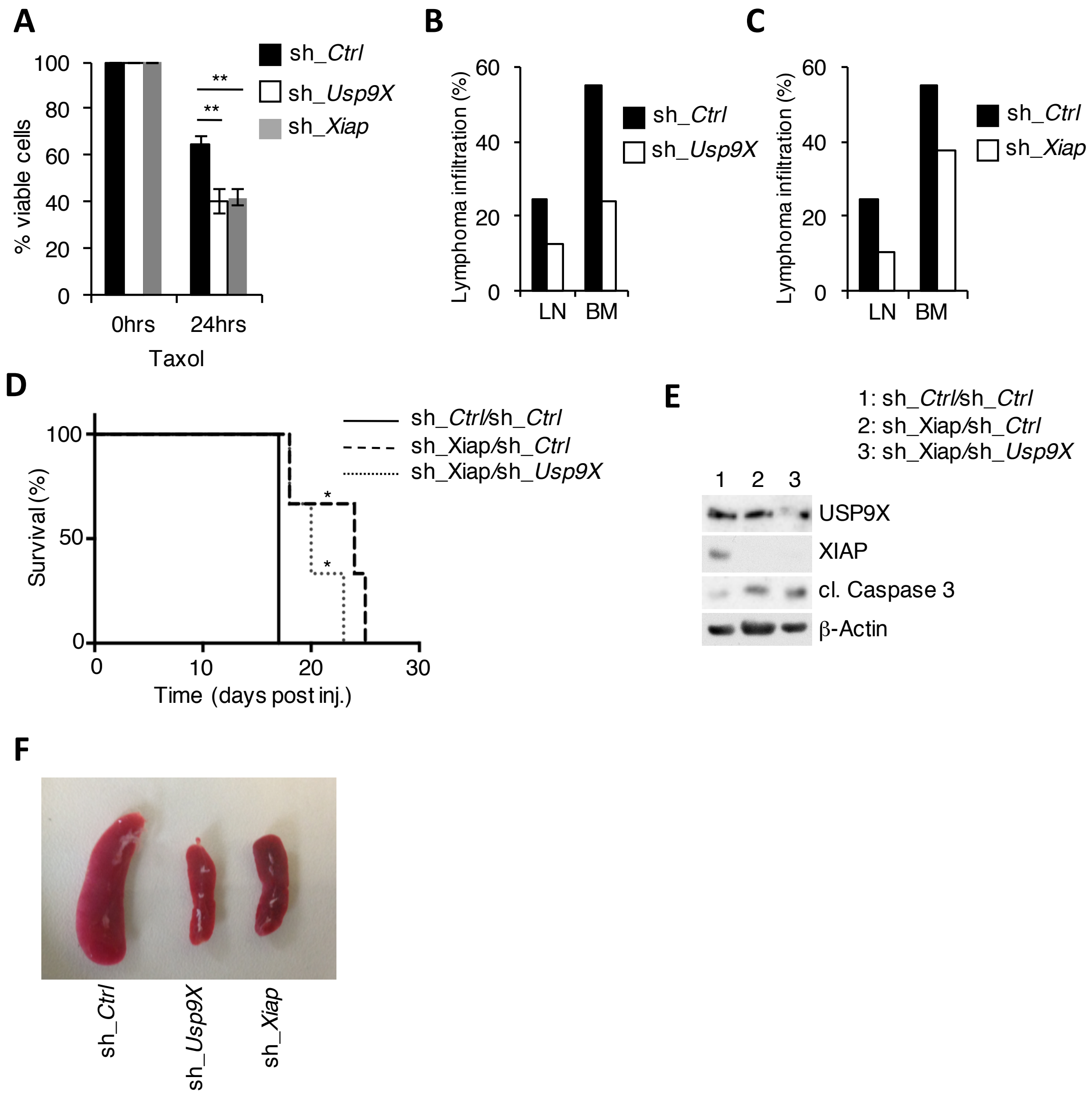

Appendix Figure S2 - Silencing of *Usp9X* and *Xiap* reduces lymphoma infiltration and treatment resistance *in vivo*

A Taxol exposure of FACS sorted (GFP$^+$ PI$^-$) Eμ-Myc lymphoma cells infected with the indicated shRNA constructs and survival rates according to relative amount of GFP$^+$ PI$^-$ cells after treatment with taxol (sh_*Ctrl* vs. sh_*Usp9X*: **, P = 0.0032; sh_*Ctrl* vs. sh_*Xiap*: ***, P = 0.0020; Student's t-test).

B Quantification of lymphoma infiltration of lymph nodes (LN) and bone marrow (BM) of mice injected with control or *Usp9X*-silenced Eμ-Myc lymphoma cells as determined by FACS analyses of GFP$^+$ PI$^-$ cells. The median values of 6 mice for shRNA *Ctrl* and 5 mice for shRNA *Usp9X* are shown.

C Quantification of lymphoma infiltration of lymph nodes (LN) and bone marrow (BM) of mice injected with control or *Xiap*-silenced Eμ-Myc lymphoma cells as described in (**B**). The median values of 6 mice per group are shown.

D Kaplan-Meier survival curves of mice that were injected with syngeneic Eμ-Myc lymphoma cells that were transduced with the indicated shRNA constructs and then sorted for double positives (sh_*Ctrl*/sh_*Ctrl*, black, n = 2; sh_*XIAP*/sh_*Ctrl*, broken black, n = 3; sh_*XIAP*/sh_*Usp9X*, dotted gray, n = 3). *, P = 0.0455; Mantel-Cox test.

E Representative immunoblot analysis of spleen tissue derived from diseased mice shown in (**D**).

F Exemplary spleen size of mice injected with Eμ-Myc lymphoma cells modified as indicated three days after intraperitoneal injection of vincristine. All three mice are at the time of sacrifice on day 18 after injection of lymphoma cells.

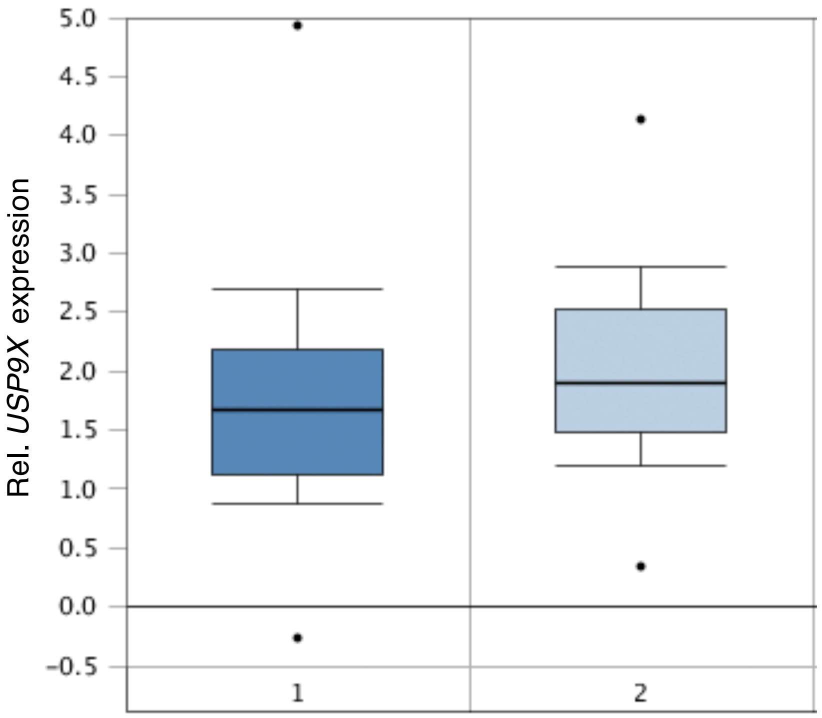

1. Activated B-Cell-Like (ABC) Diffuse Large B-Cell Lymphoma (n = 167)
2. Germinal Center B-Cell-Like (GCB) Diffuse Large B-Cell Lymphoma (n = 183)

Appendix Figure S3 – USP9X gene expression in ABC- and GCB-type DLBCL

Data from (Lenz et al, 2008) reanalyzed to show expression levels of *USP9X* in ABC (1) and GCB (2) subtypes of DLBCL with non-significant difference between USP9X expression in ABC and GCB-subtype (P = 0.282). Box-and-whisker plots show the upper and lower quartiles (25–75%) with a line at the median, whiskers extend from the 10th to the 90th percentile, and dots correspond to minimal and maximal values. Data was provided by the Oncomine database.

	RICOVER-60<br>n=1222		
	Patients with TMA material<br>n=121	Patients without TMA material<br>n=1101	p
Male	59 (49%)	591 (54%)	0.303
Age, median (range)	69 (61-79)	68 (61-80)	0.695
LDH>UNV	57 (47%)	547 (50%)	0.591
ECOG >1	15 (12%)	161 (15%)	0.508
Stage III/IV	55 (46%)	564 (51%)	0.228
Extranodal sites >1	17 (14%)	199 (18%)	0.271
IPI 1	42 (35%)	330 (30%)	0.679
IPI 2	34 (28%)	305 (28%)	
IPI 3	28 (23%)	285 (26%)	
IPI 4,5	17 (14%)	181 (16%)	

Appendix Table S1 – Patient characteristics

Patient characteristics of the 121 TMA samples analyzed in Fig. 4 F and G in comparison to the full patient population of the RICOVER-60 trial indicating representativeness of the analyzed population.
