# Supplementary material for: USP9X stabilizes XIAP to regulate mitotic cell death and chemoresistance in aggressive B‐cell lymphoma
Source: EMBO Mol Med. 2016 Jun 17;8(8):851–62. doi: 10.15252/emmm.201506047 (PMC4967940; doi:10.15252/emmm.201506047)
Supplement: Supplementary file 2 — Expanded View Figures PDF [file EMMM-8-851-s002.pdf]

Expanded View Figures

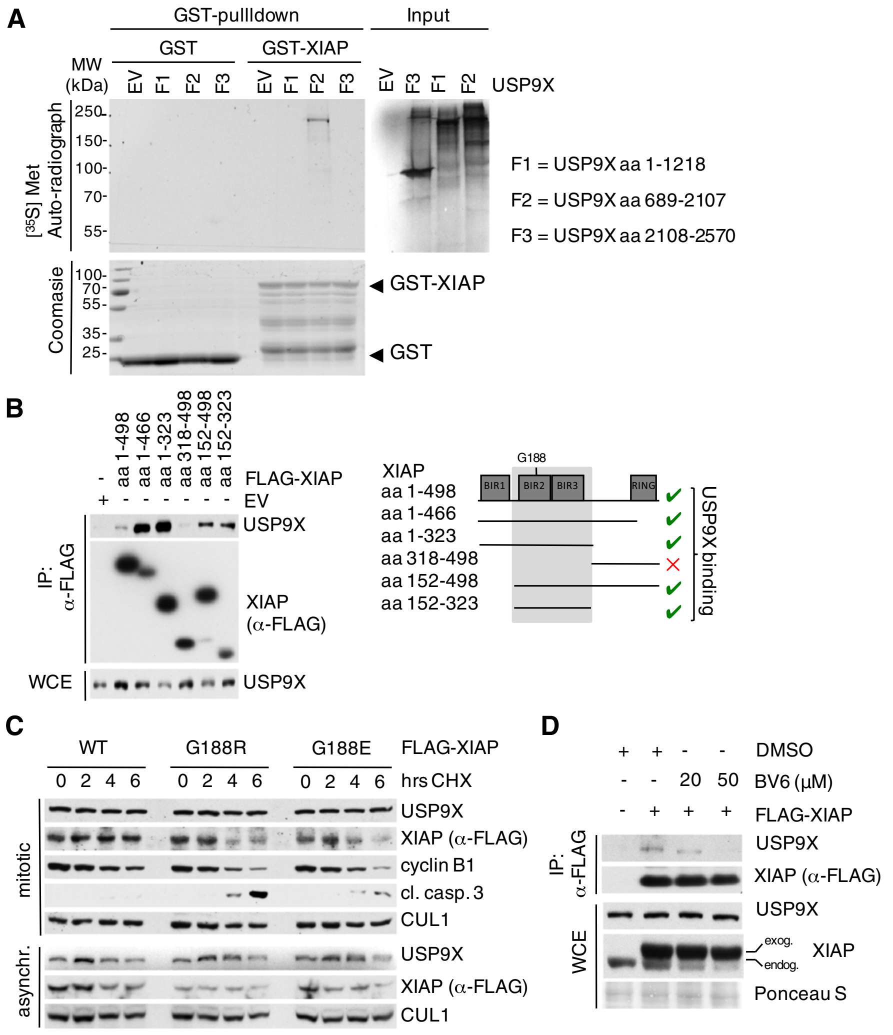

Figure EV1. USP9X interacts with XIAP in a direct manner and its active site binds to the BIR2 domain of XIAP via glycine 188.

- A *In vitro* co-immunoprecipitation of GST-purified XIAP with *in vitro* translated fragments of human USP9X with F2 containing the active site (aa 1556–1902).
- B Co-immunoprecipitation of either full-length or different fragments of FLAG-tagged XIAP with endogenous USP9X from HEK 293T cells that were transfected with the indicated expression constructs and synchronized in mitosis using nocodazole.
- C Immunoblot analyses of HeLa cells that were transfected with the indicated WT and mutant XIAP expression constructs and treated with cycloheximide (CHX) for the times specified.
- D Co-immunoprecipitation of FLAG-tagged XIAP with endogenous USP9X from HEK 293T cells that were treated with BV6 as specified and nocodazole for 12 h.

Source data are available online for this figure.

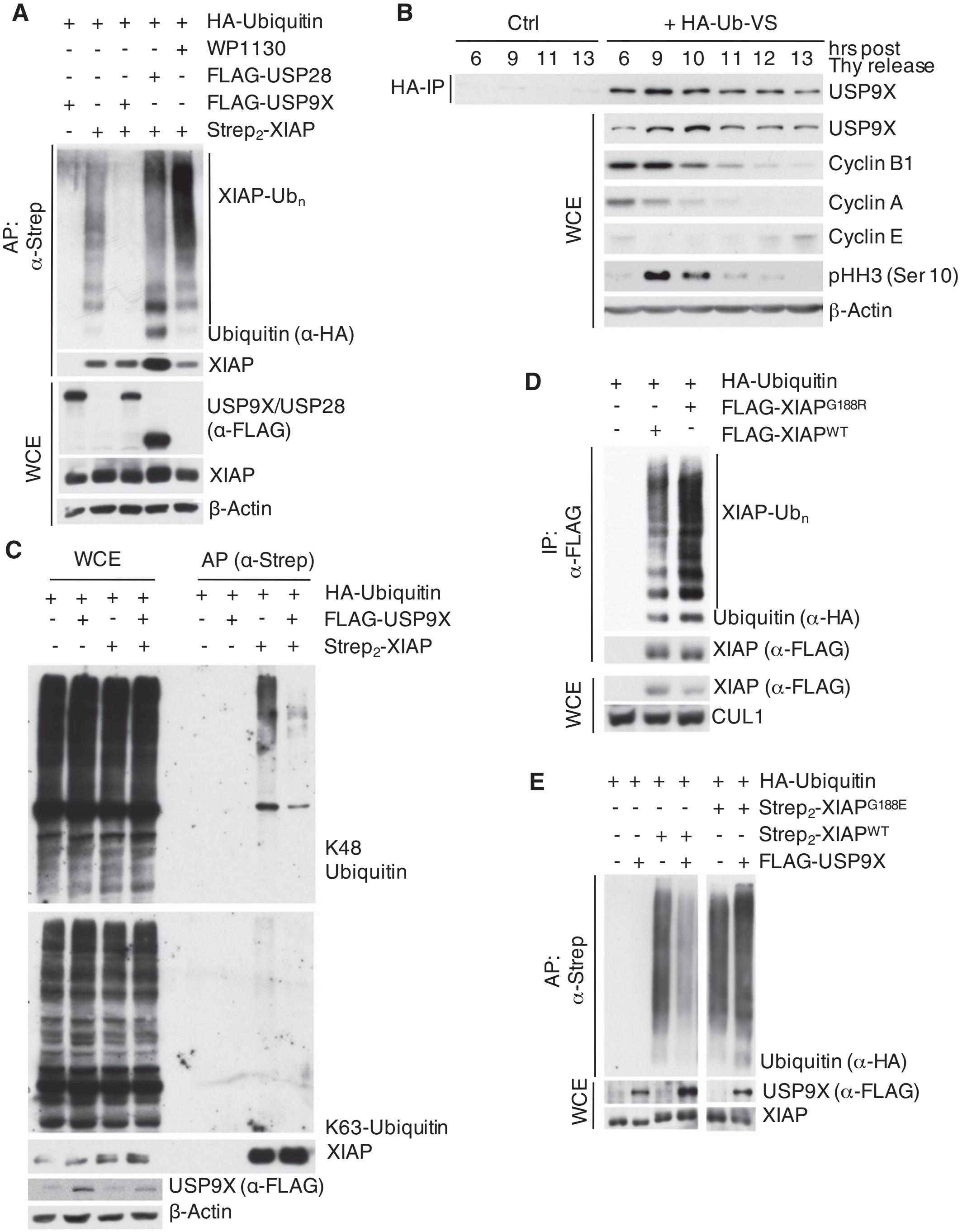

Figure EV2. USP9X deubiquitylates XIAP-WT, but not XIAP-G188R or XIAP-G188E, in mitosis.

A *In vivo* ubiquitylation of XIAP in HEK 293T cells that were co-transfected with the indicated expression constructs, synchronized in mitosis using nocodazole, and treated with MG132 prior to harvesting. The USP9X inhibitor WP1130 was added for 2 h as specified. XIAP was isolated by streptavidin affinity purification (AP) using denaturing conditions.

B HeLa cells were arrested in S phase with double thymidine block, released, and collected at the indicated time points. Deubiquitination activity was assessed by addition of HA-tagged dominant negative diubiquitin and following HA-IP under denaturing conditions.

C Immunoblot analysis of *in vivo* ubiquitylated XIAP (prepared as in A) using K48- or K63-specific ubiquitin antibodies.

D *In vivo* ubiquitylation of $XIAP^{WT}$ or $XIAP^{G188R}$ in HEK 293T cells that were co-transfected with the indicated expression constructs, synchronized in mitosis, and treated with MG132 as in (A). $XIAP^{WT}$ or $XIAP^{G188R}$ were isolated by anti-FLAG immunoprecipitation under denaturing conditions.

E *In vivo* ubiquitylation of $XIAP^{WT}$ or $XIAP^{G188E}$ in HEK 293T cells that were co-transfected with the indicated expression constructs and treated as in (A). $XIAP^{WT}$ or $XIAP^{G188E}$ were isolated by streptavidin affinity purification under denaturing conditions.

Source data are available online for this figure.

 © 2016 The Authors

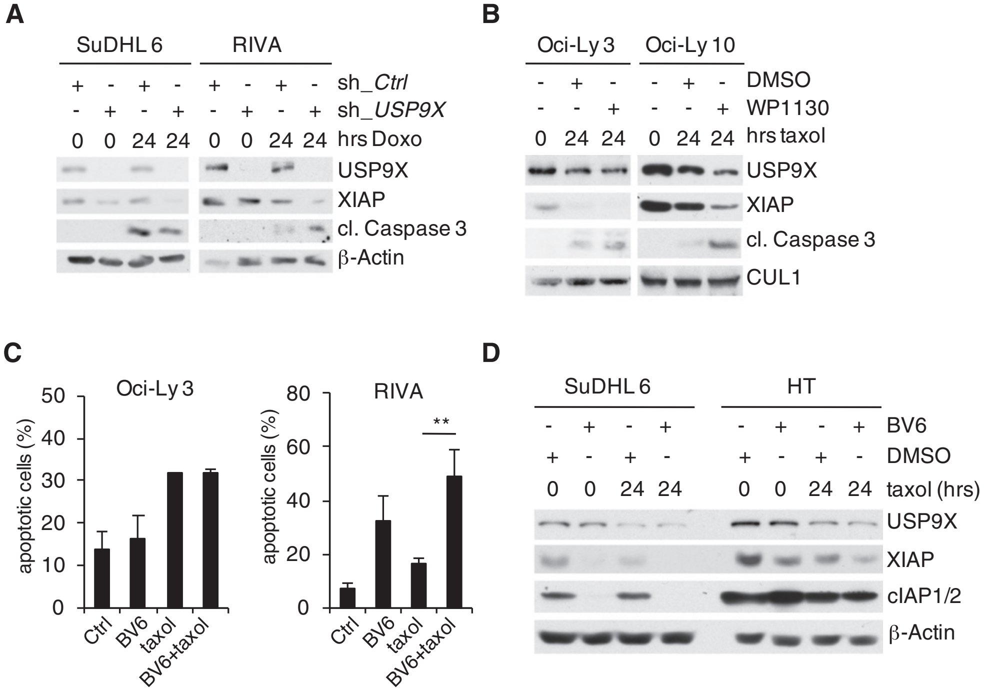

Figure EV3. Mitotic stabilization of XIAP by USP9X mediates resistance to spindle poisons.

A Immunoblot analyses of the indicated DLBCL cell lines that were lentivirally transduced with IRES-GFP shRNA constructs against *USP9X* or a non-relevant mRNA, FACS sorted for GFP$^+$ PI$^-$ cells and exposed to doxorubicin for the indicated periods of time.

B Immunoblot analyses of the indicated DLBCL cell lines that were exposed to taxol for the indicated periods of time. Two hours before collecting, WP1130 at a concentration of 5 μM or DMSO was added as specified.

C FACS analysis (propidium iodide (PI) uptake) of DLBCL cell lines treated with taxol and/or the SMAC mimetic BV6 as indicated. Results displayed are from three independent experiments each (n = 3, ± SD). **P = 0.00643; Student's t-test.

D Immunoblot analyses of the indicated DLBCL cell lines that were treated with taxol or the SMAC mimetic BV6 as indicated.

Source data are available online for this figure.
