# Supplementary material for: USP9X stabilizes XIAP to regulate mitotic cell death and chemoresistance in aggressive B‐cell lymphoma
Source: EMBO Mol Med. 2016 Jun 17;8(8):851–62. doi: 10.15252/emmm.201506047 (PMC4967940; doi:10.15252/emmm.201506047)

A

Extended View Fig. 3

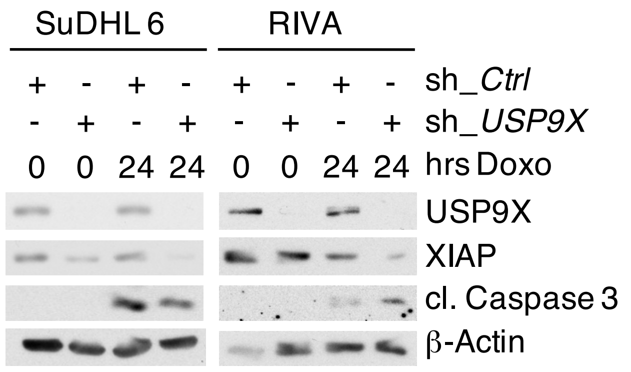

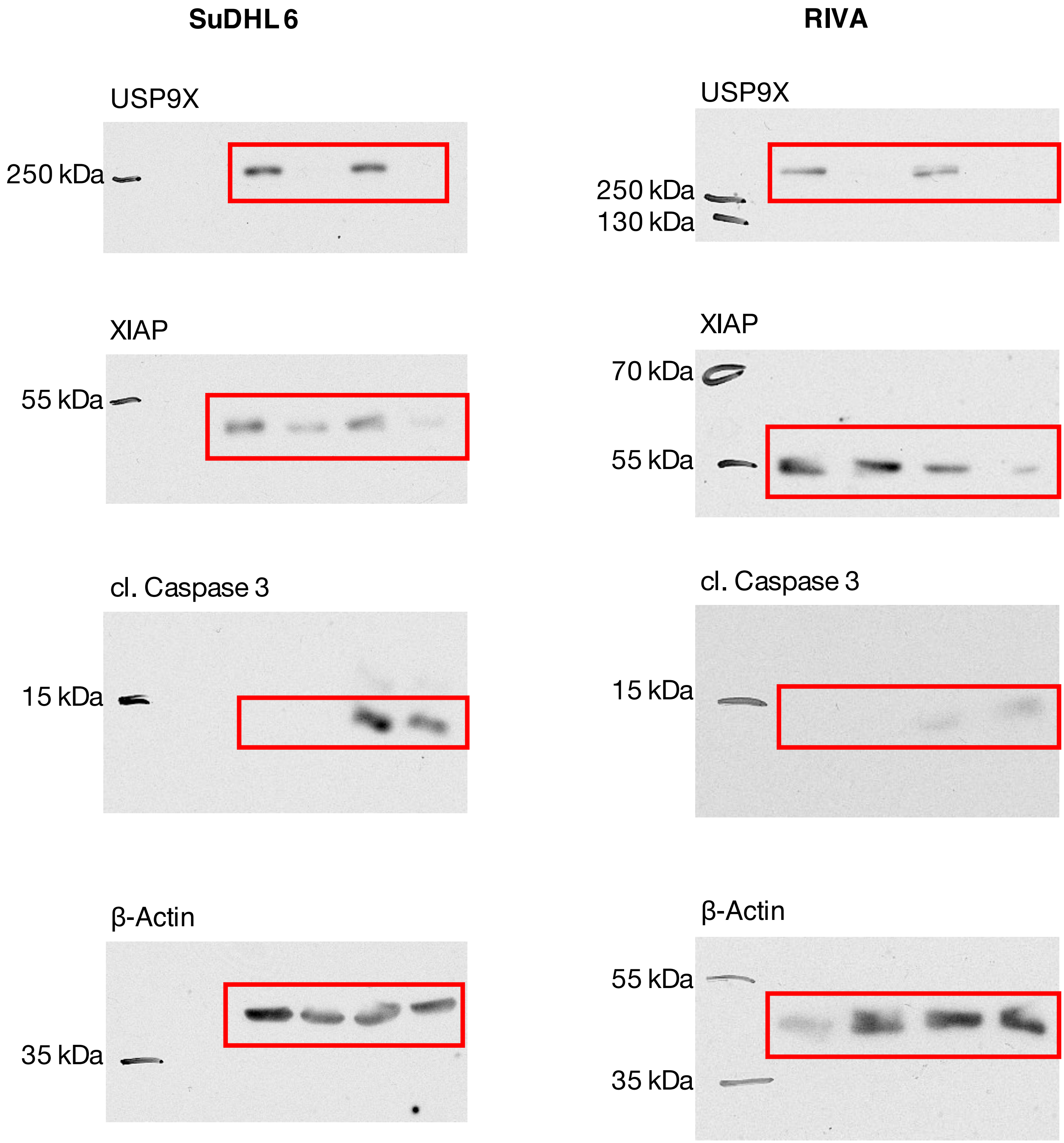

Extended View Fig. 3

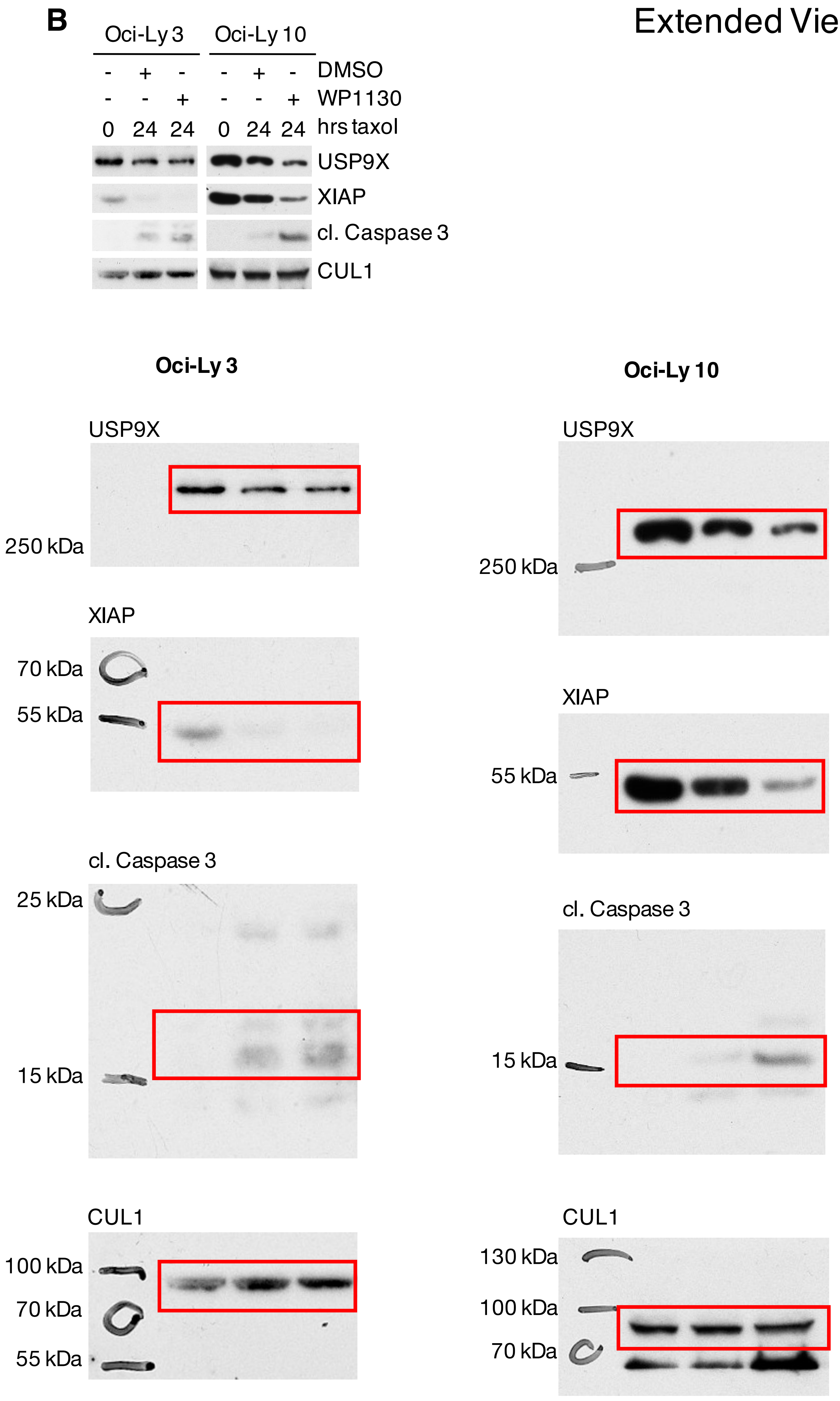

Extended View Fig. 3

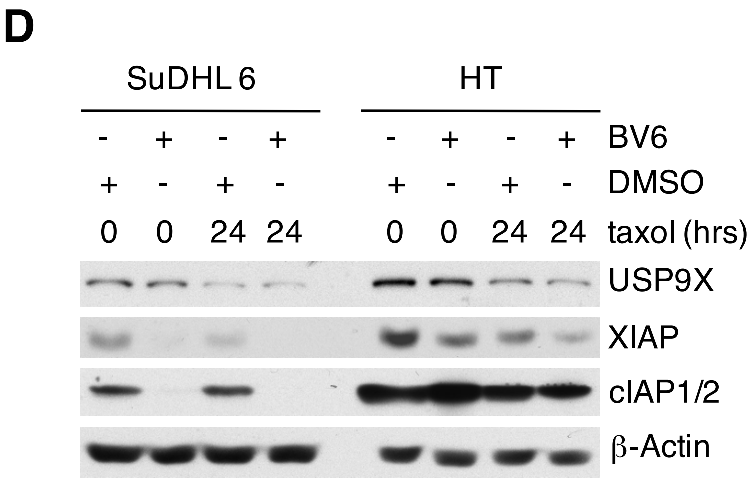

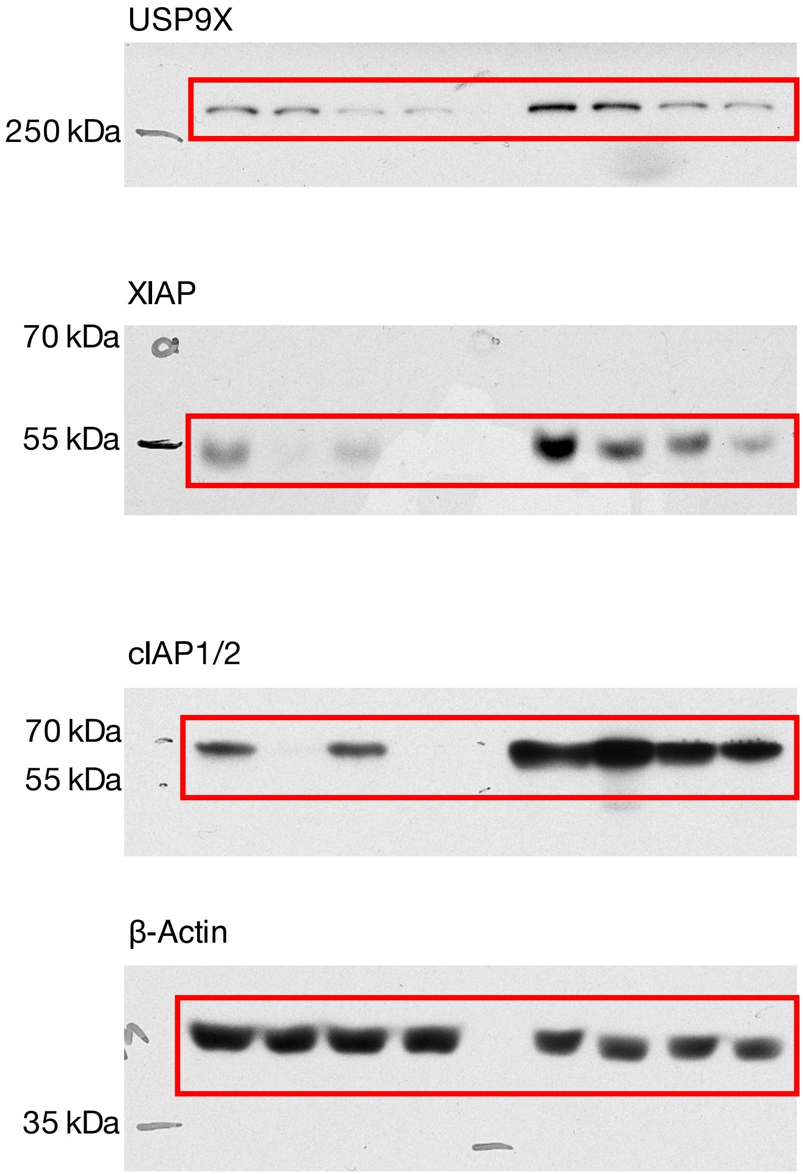

Supplement: Supplementary file 3 — Source Data for Expanded View and Appendix [file EMMM-8-851-s003.zip › Source_Data_for_Appendix_and_Expanded_View/Source_data_EV_figure_3.pdf]
