# Supplementary figures and images for: USP9X stabilizes XIAP to regulate mitotic cell death and chemoresistance in aggressive B‐cell lymphoma
Source: EMBO Mol Med. 2016 Jun 17;8(8):851–62. doi: 10.15252/emmm.201506047 (PMC4967940; doi:10.15252/emmm.201506047)

Figure Appendix S2

**E**

1: sh\_Ctrl/sh\_Ctrl  
2: sh\_Xiap/sh\_Ctrl  
3: sh\_Xiap/sh\_Usp9X

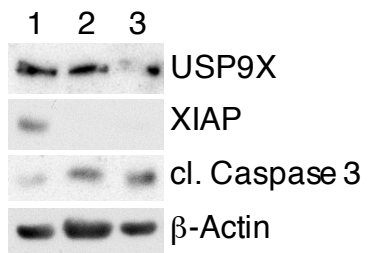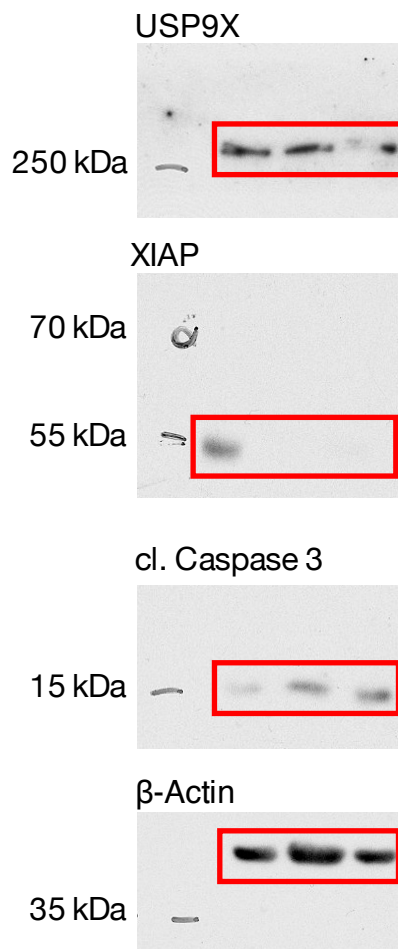

Supplement: Supplementary file 3 — Source Data for Expanded View and Appendix [file EMMM-8-851-s003.zip › Source_Data_for_Appendix_and_Expanded_View/Source_data_appendix_figure_S2.pdf]

# Extended View Fig. 1

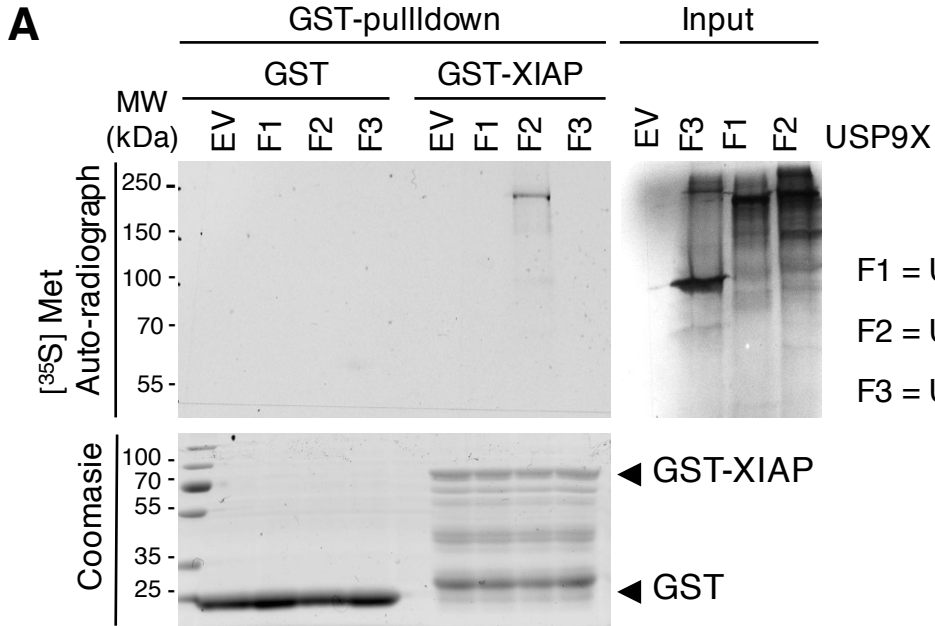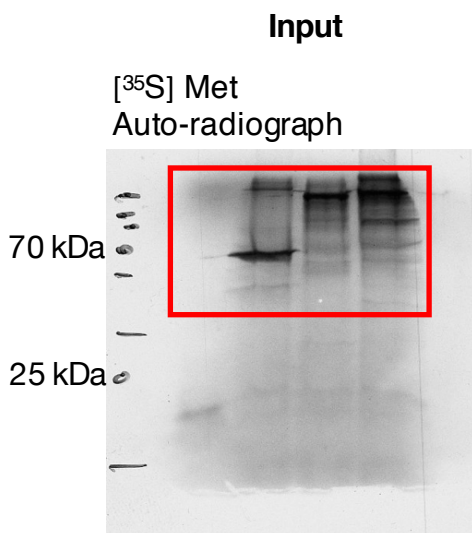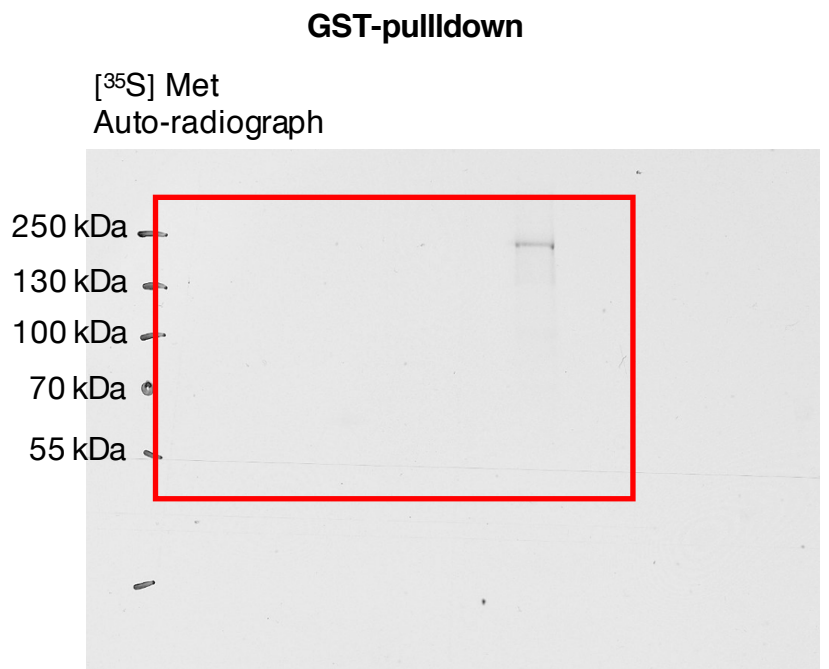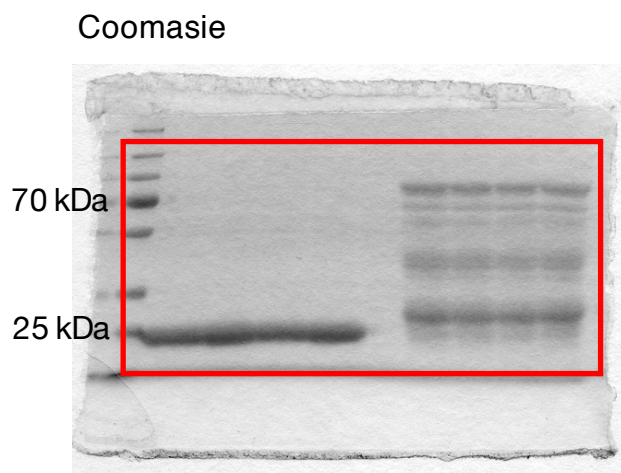

**B**

Extended View Fig. 1

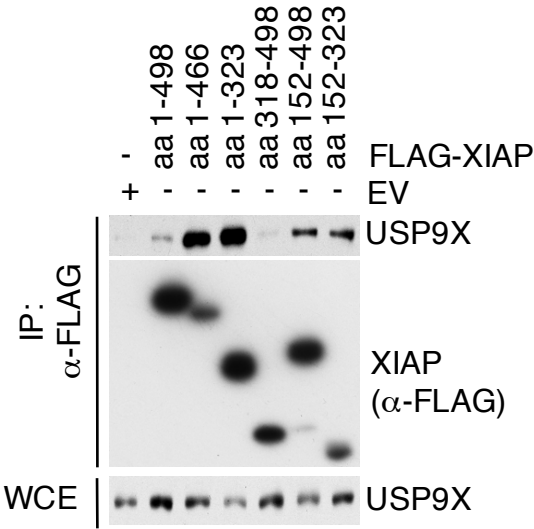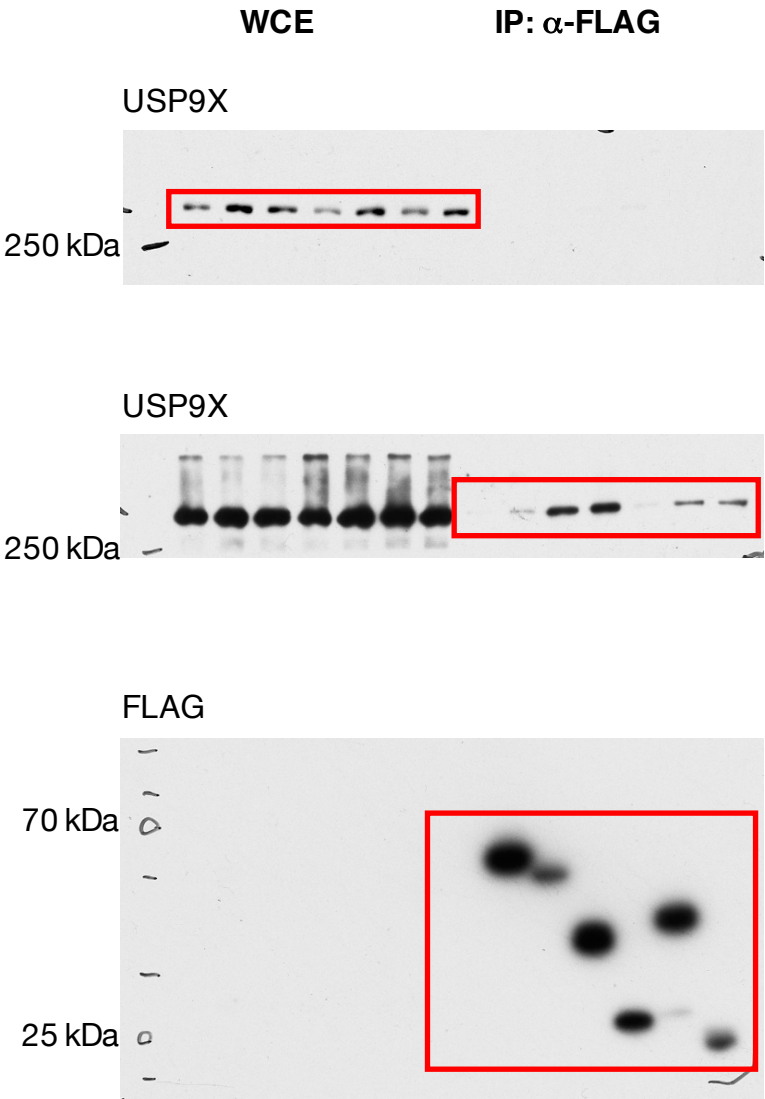

**C**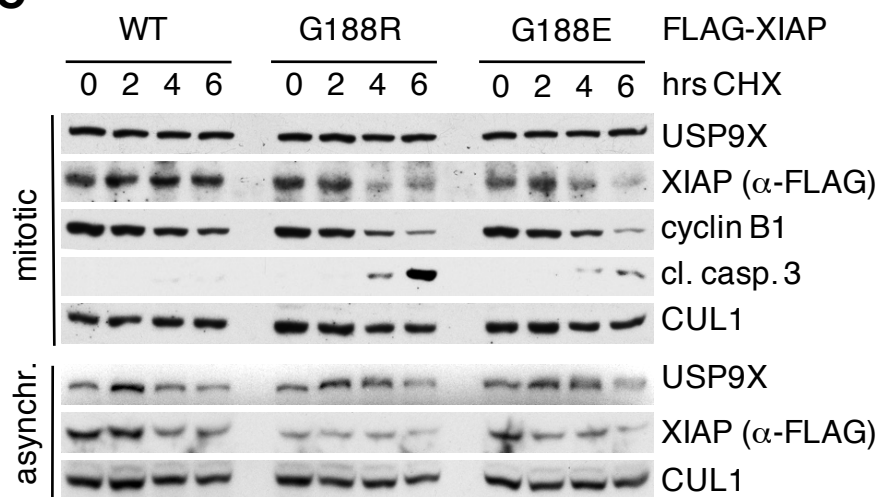

Extended View Fig. 1

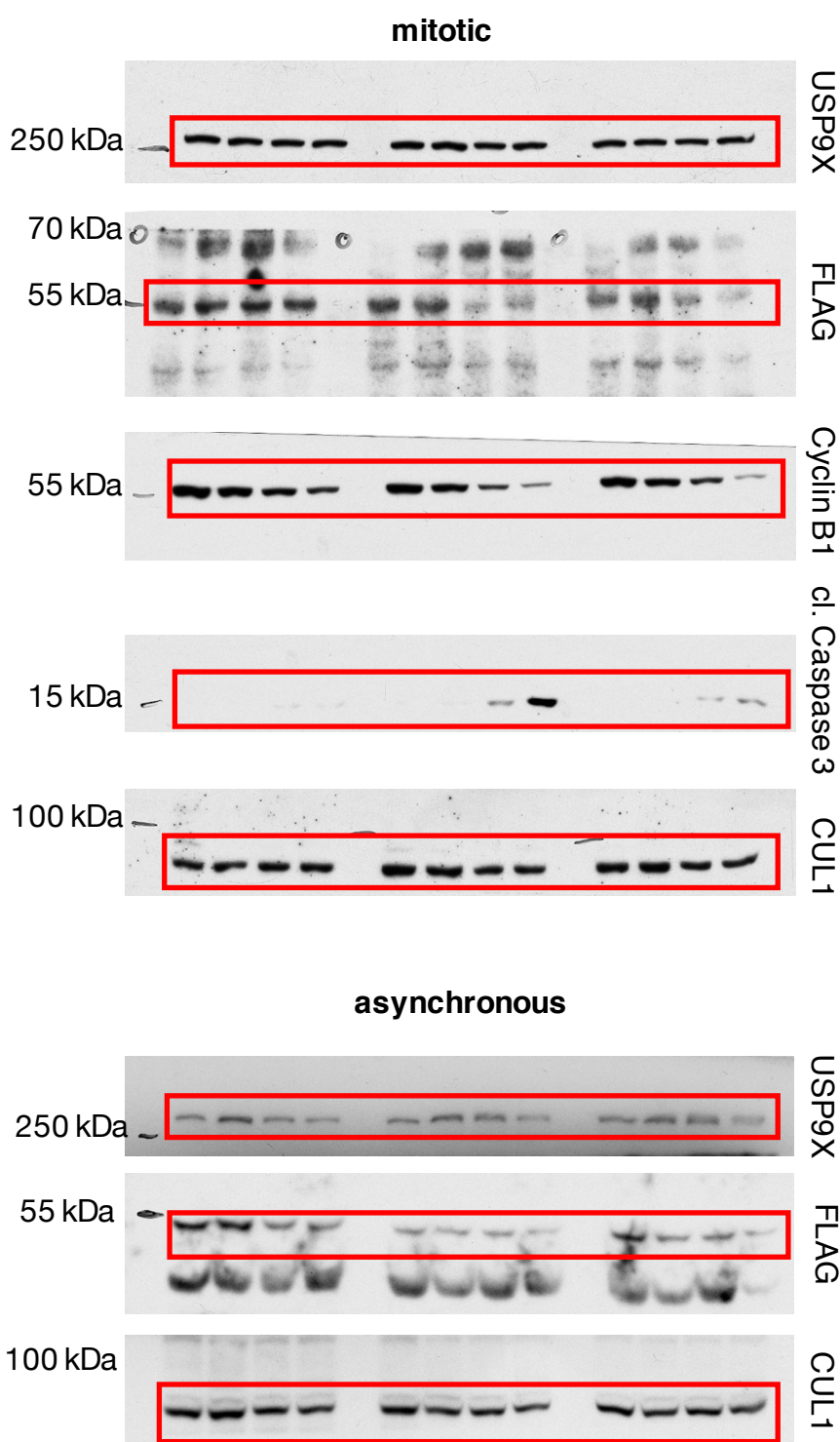

**D**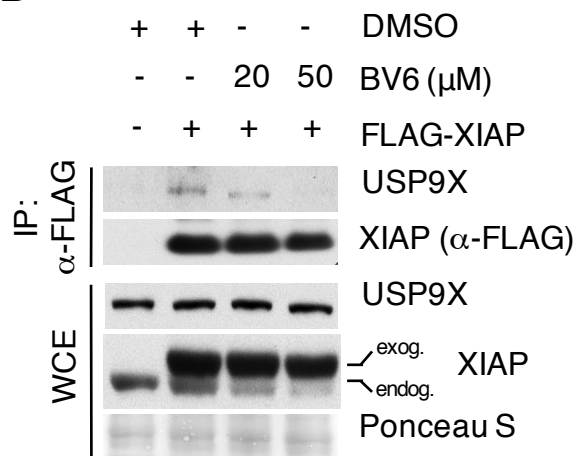

Extended View Fig. 1

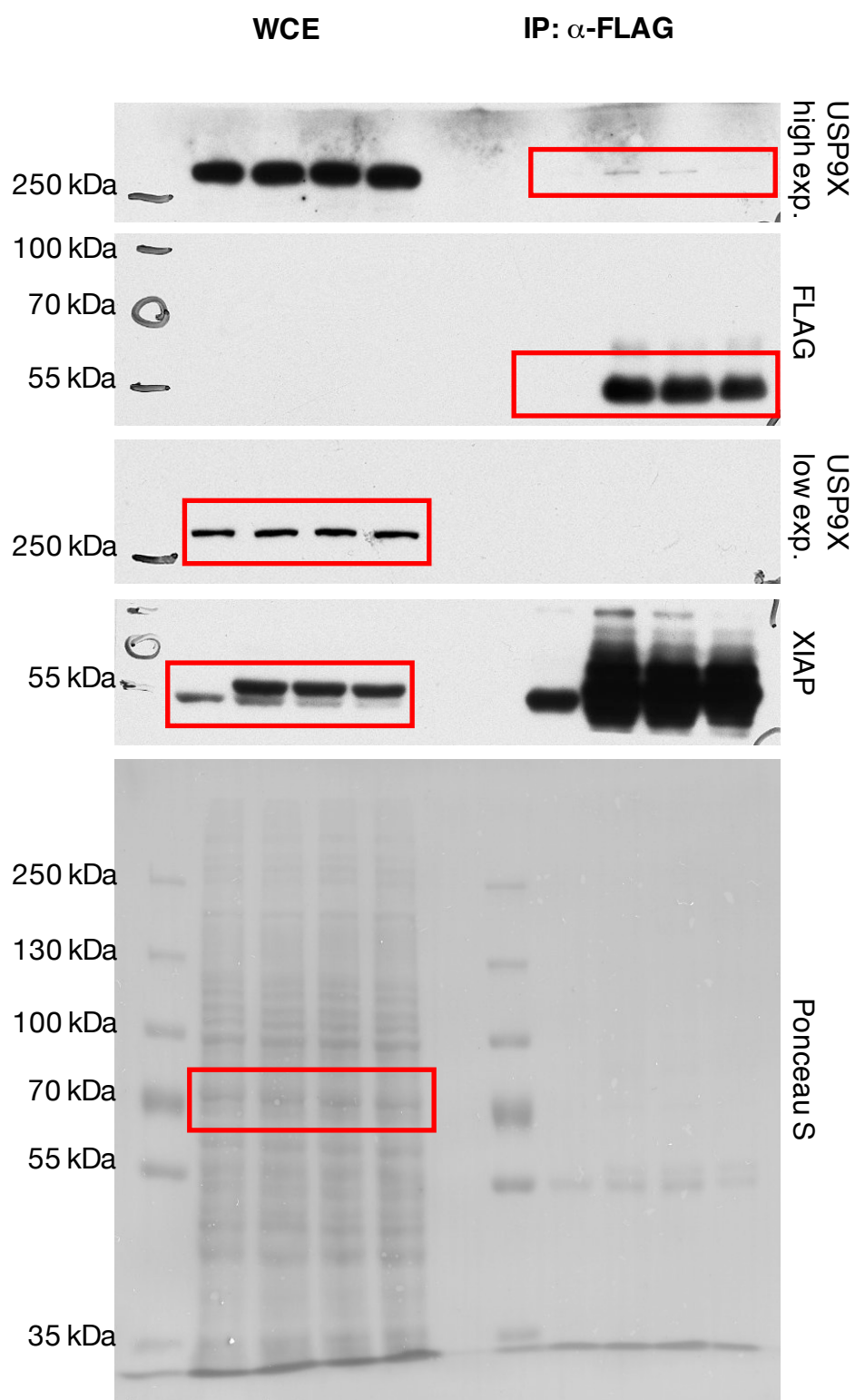

Supplement: Supplementary file 3 — Source Data for Expanded View and Appendix [file EMMM-8-851-s003.zip › Source_Data_for_Appendix_and_Expanded_View/Source_data_EV_figure_1.pdf]

Figure 3

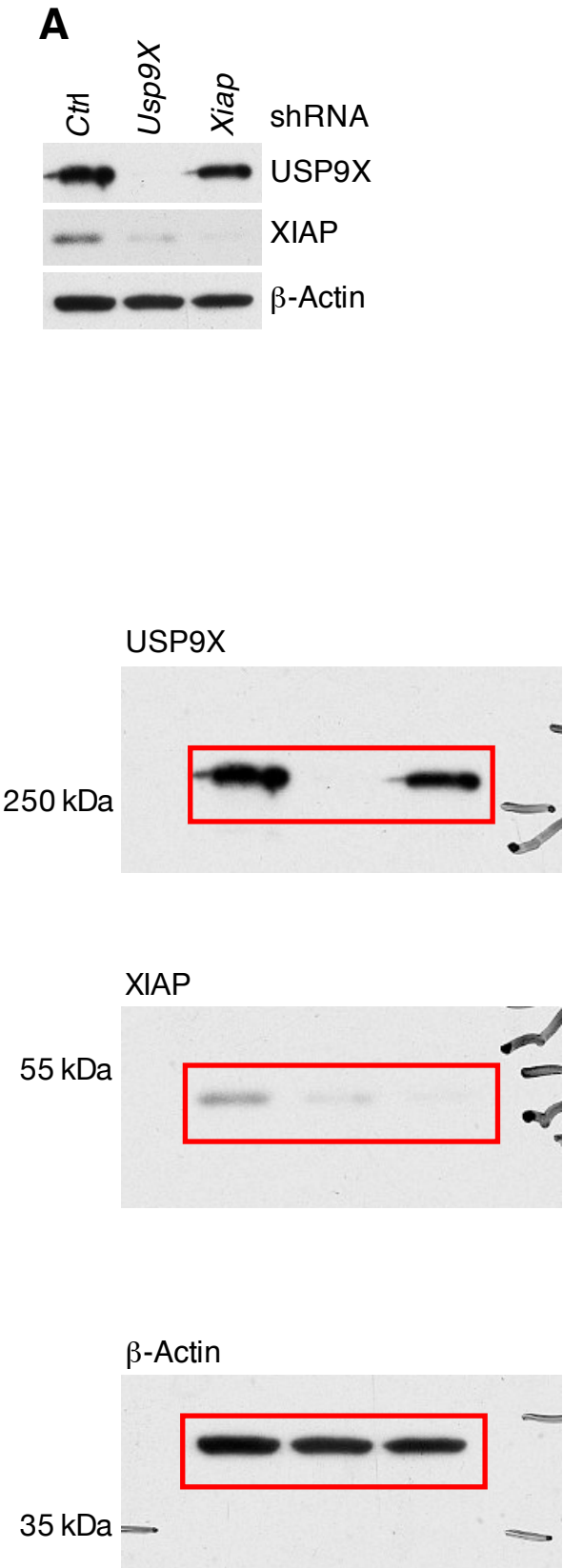

Supplement: Supplementary file 7 — Source Data for Figure 3 [file EMMM-8-851-s006.pdf]

Figure 4

**A**

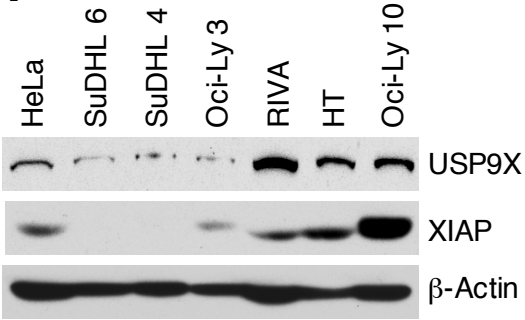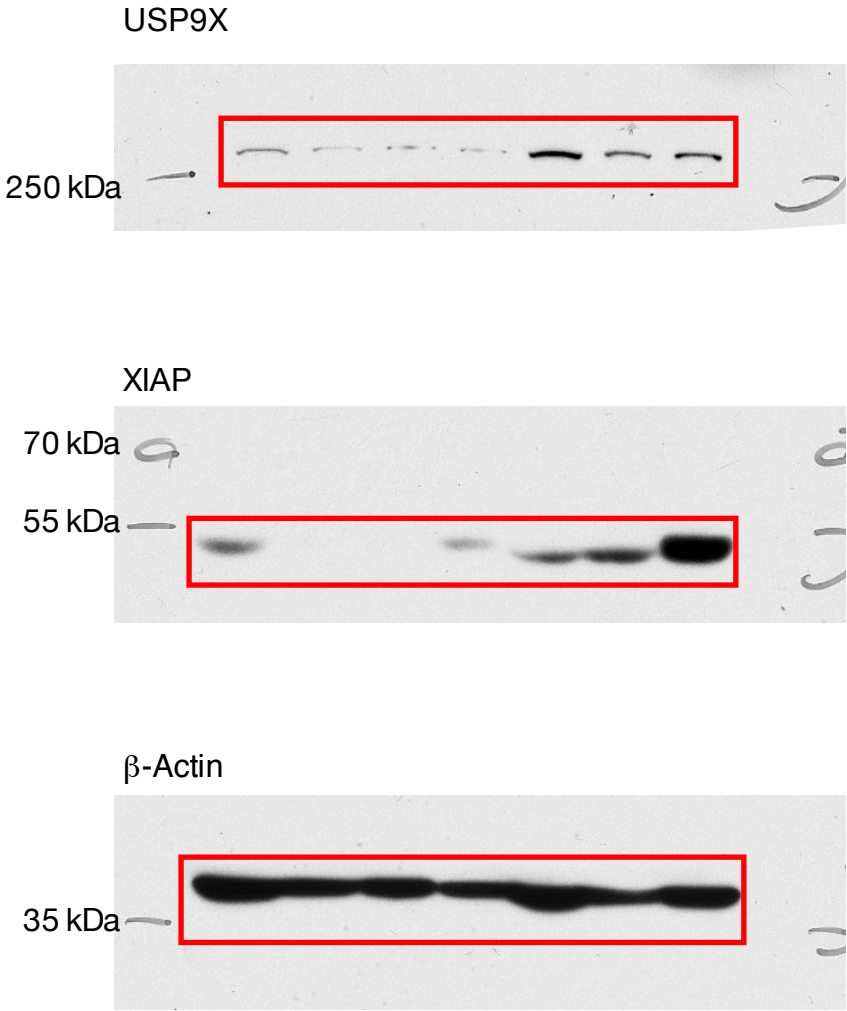

C

Figure 4

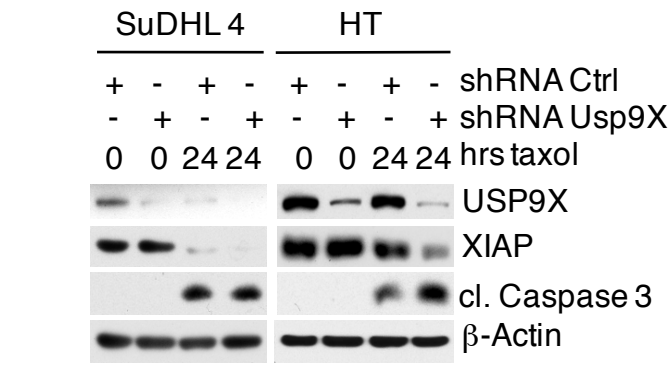

SuDHL 4

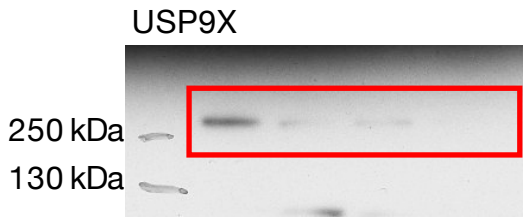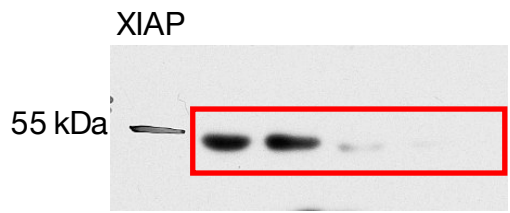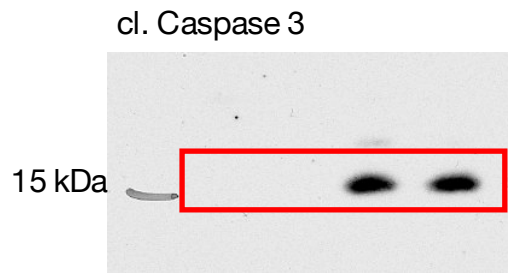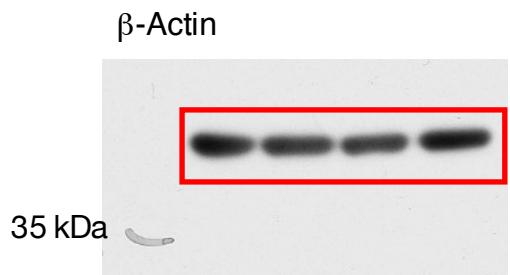

HT

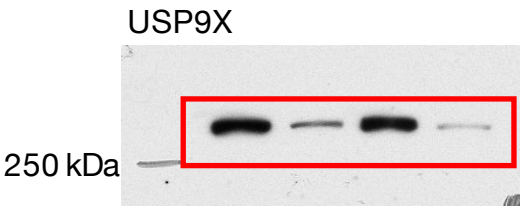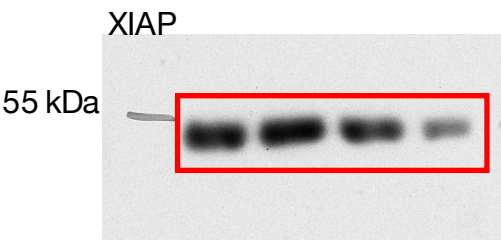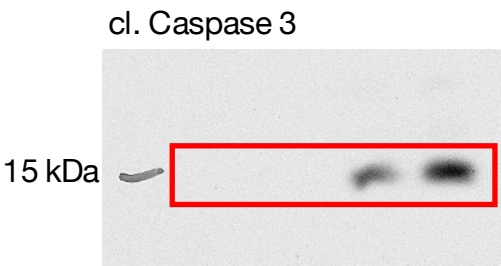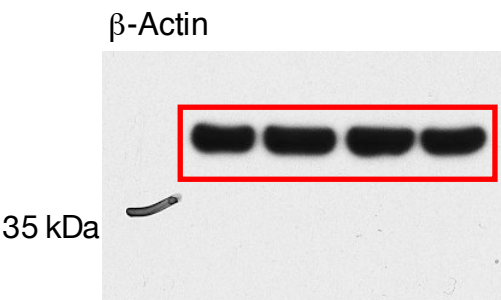

Figure 4

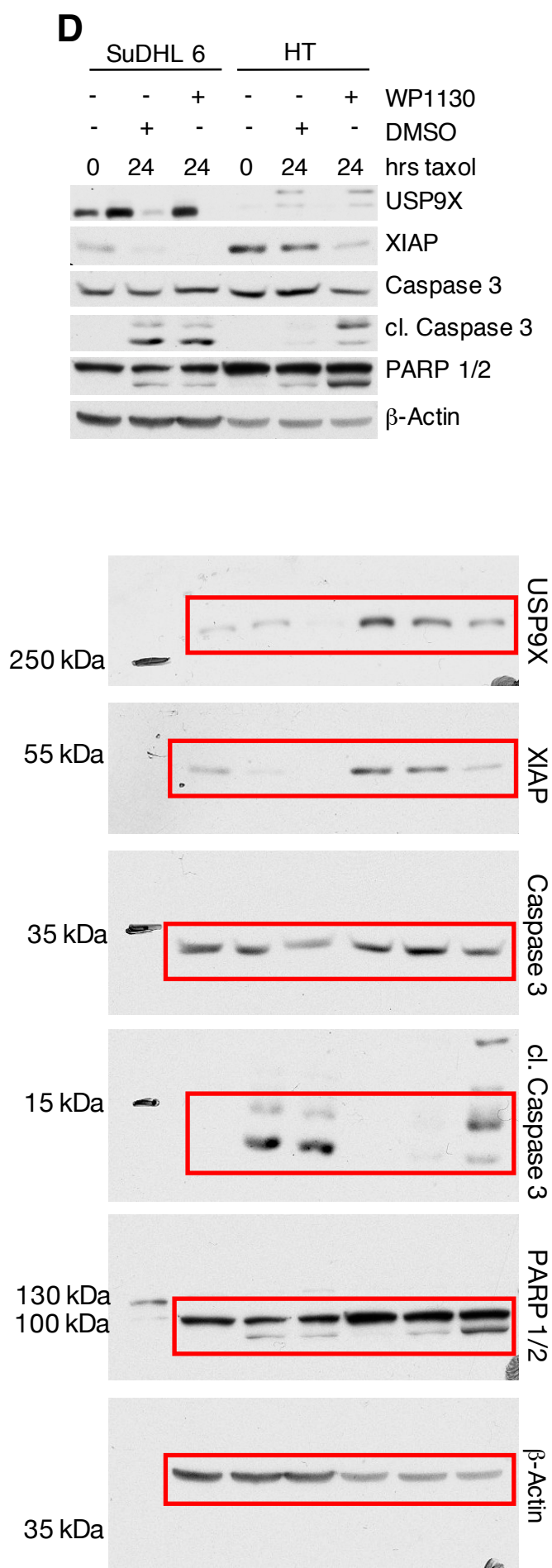

Supplement: Supplementary file 8 — Source Data for Figure 4 [file EMMM-8-851-s007.pdf]
