# Supplementary material for: USP9X stabilizes XIAP to regulate mitotic cell death and chemoresistance in aggressive B‐cell lymphoma
Source: EMBO Mol Med. 2016 Jun 17;8(8):851–62. doi: 10.15252/emmm.201506047 (PMC4967940; doi:10.15252/emmm.201506047)
Supplement: Supplementary file 5 — Source Data for Figure 1 [file EMMM-8-851-s004.pdf]

Figure 1

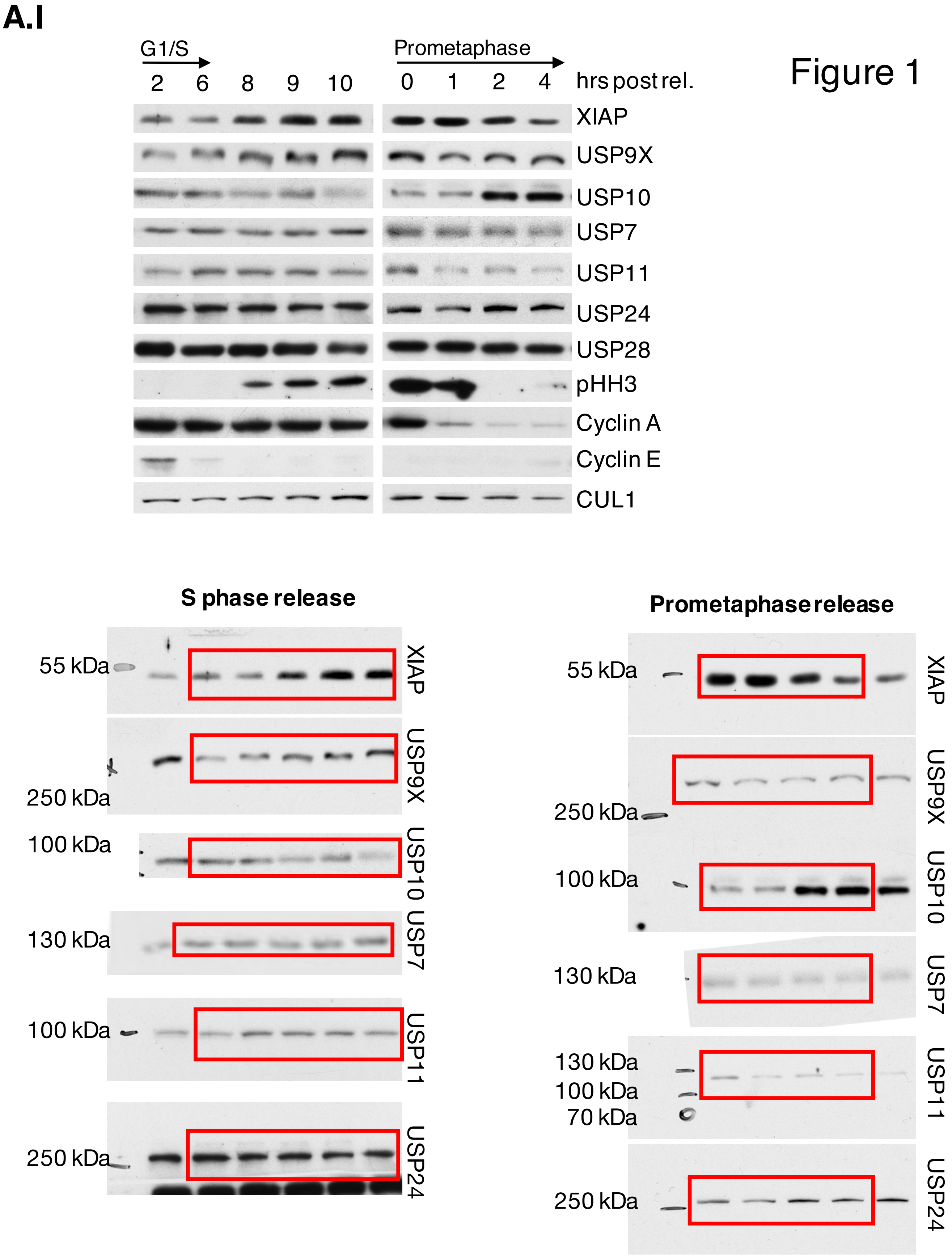
A.I
G1/S
2
6
8
9
10
Prometaphase
0
1
2
4
hrs post rel.
XIAP
USP9X
USP10
USP7
USP11
USP24
USP28
pHH3
Cyclin A
Cyclin E
CUL1
S phase release
55 kDa
250 kDa
100 kDa
130 kDa
100 kDa
250 kDa
XIAP
USP9X
USP10
USP7
USP11
USP24
Prometaphase release
55 kDa
250 kDa
100 kDa
130 kDa
130 kDa
100 kDa
70 kDa
250 kDa
XIAP
USP9X
USP10
USP7
USP11
USP24

A.II

Figure 1

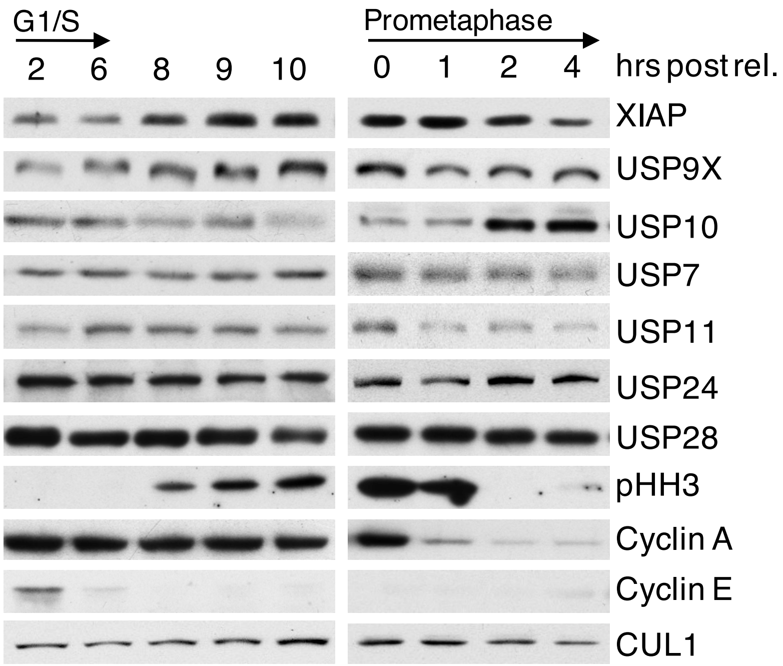

G1/S
Prometaphase
2 6 8 9 10
0 1 2 4
hrs post rel.
XIAP
USP9X
USP10
USP7
USP11
USP24
USP28
pHH3
Cyclin A
Cyclin E
CUL1

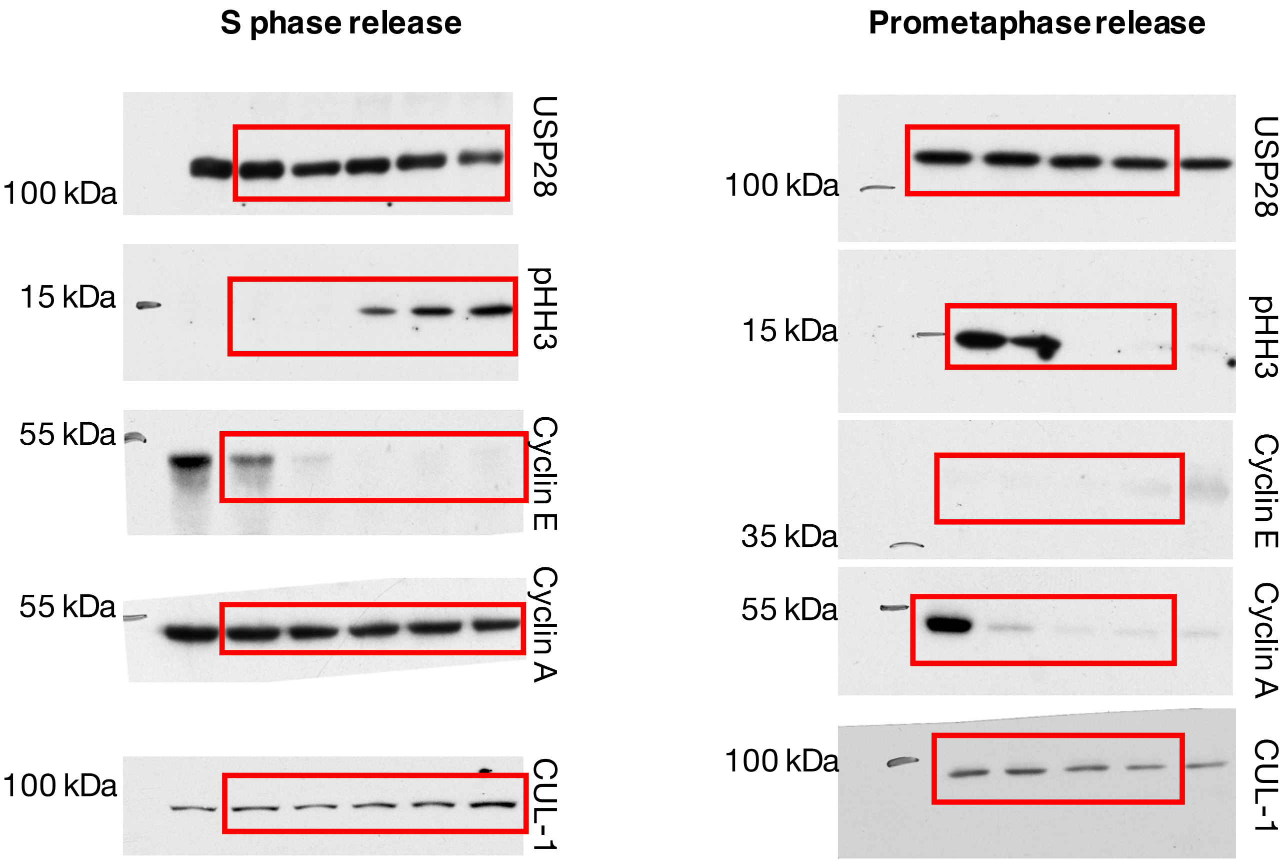

S phase release
Prometaphase release
100 kDa
USP28
15 kDa
pHH3
55 kDa
Cyclin E
35 kDa
55 kDa
Cyclin A
100 kDa
CUL-1

Figure 1

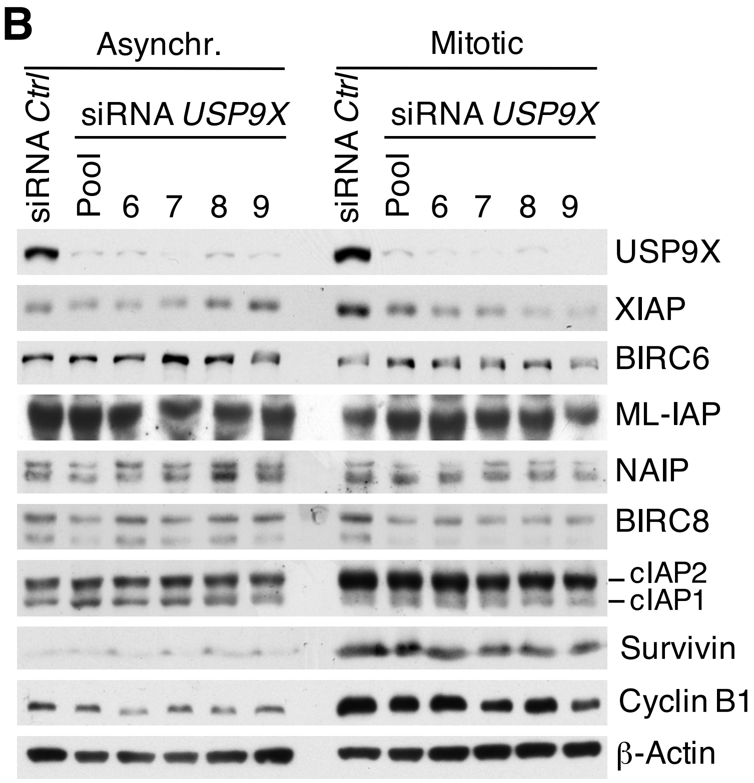

B
Asynchr.
Mitotic
siRNA Ctrl
siRNA USP9X
Pool
6
7
8
9
USP9X
XIAP
BIRC6
ML-IAP
NAIP
BIRC8
cIAP2
cIAP1
Survivin
Cyclin B1
β-Actin

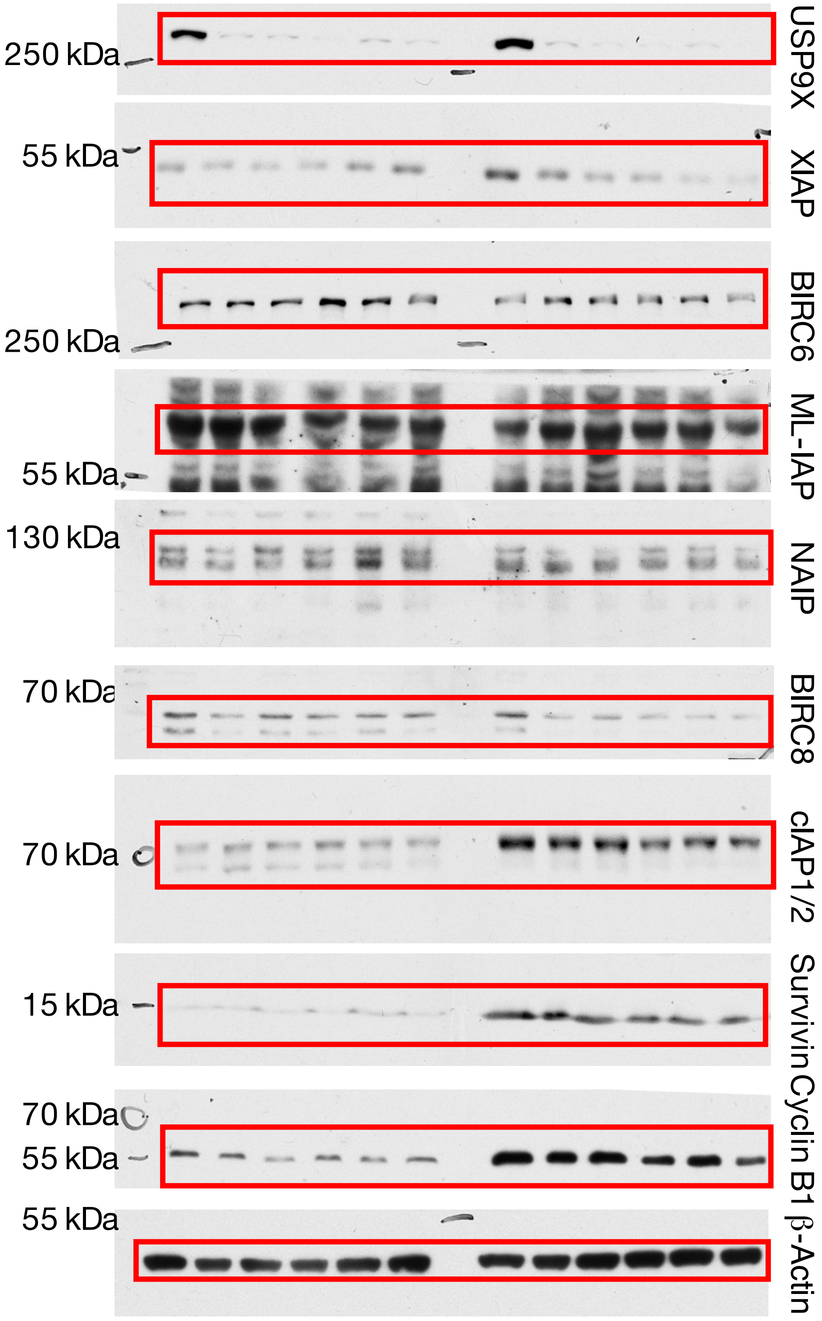

250 kDa
55 kDa
250 kDa
55 kDa
130 kDa
70 kDa
70 kDa
15 kDa
70 kDa
55 kDa
55 kDa
USP9X
XIAP
BIRC6
ML-IAP
NAIP
BIRC8
cIAP1/2
Survivin
Cyclin B1
β-Actin

Figure 1

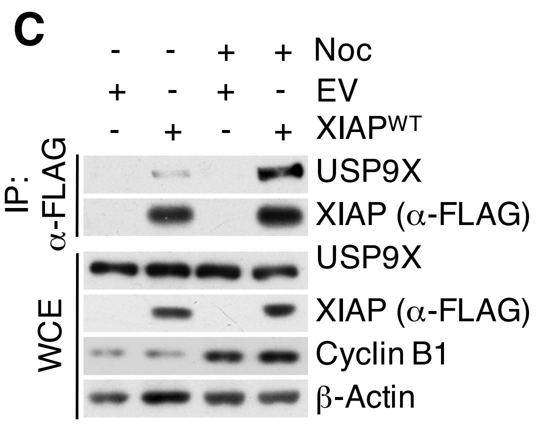
C
- - + + Noc
+ - + - EV
- + - + XIAPWT
IP: α-FLAG
USP9X
XIAP (α-FLAG)
WCE
USP9X
XIAP (α-FLAG)
Cyclin B1
β-Actin

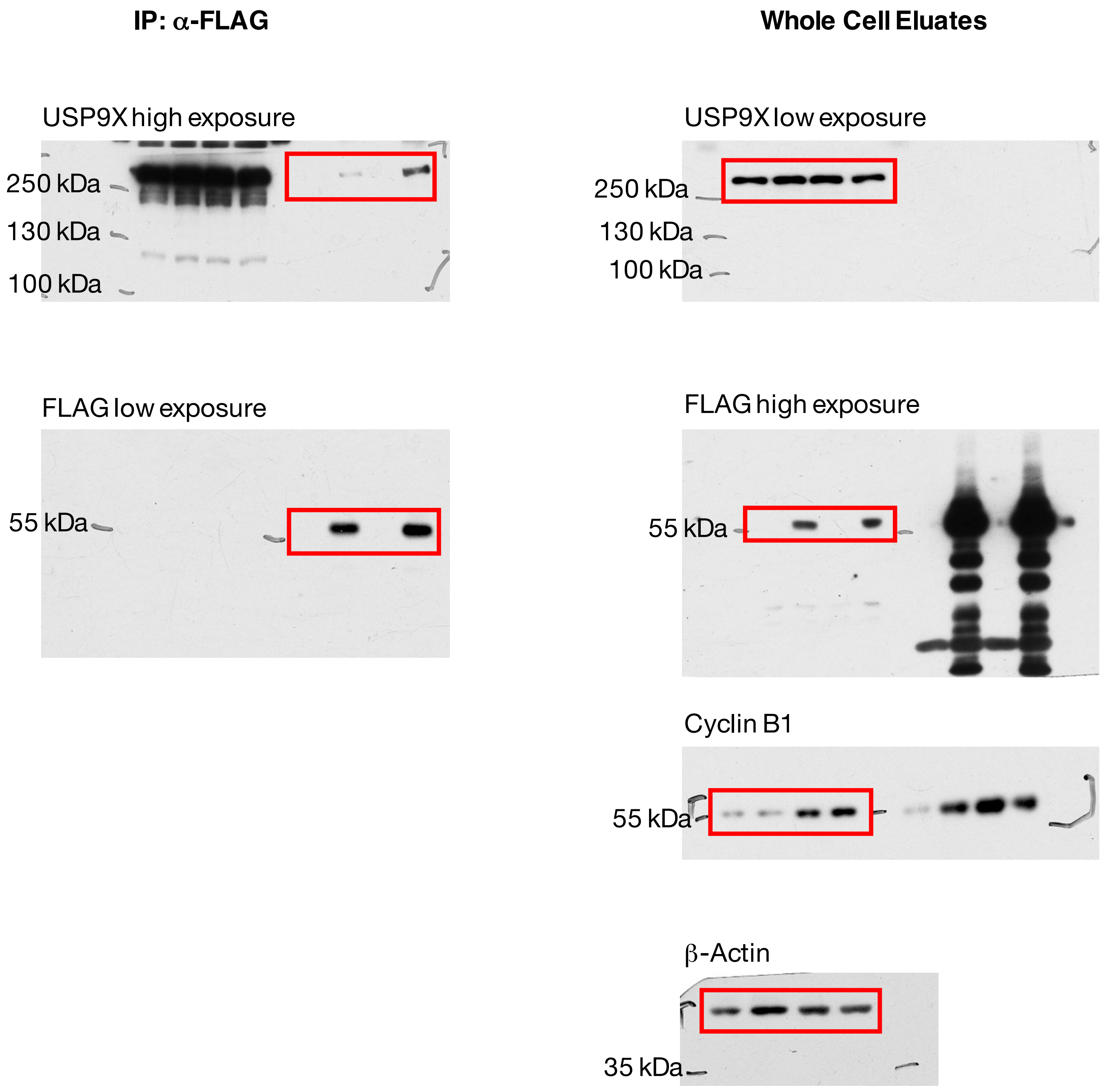
IP: α-FLAG
Whole Cell Eluates
USP9X high exposure
250 kDa
130 kDa
100 kDa
USP9X low exposure
250 kDa
130 kDa
100 kDa
FLAG low exposure
55 kDa
FLAG high exposure
55 kDa
Cyclin B1
55 kDa
β-Actin
35 kDa

Figure 1

D

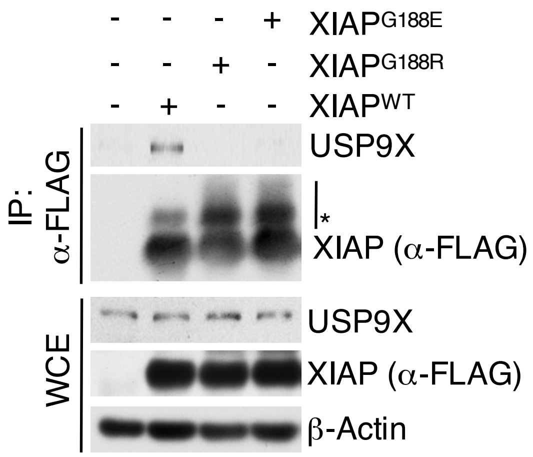

- - - + XIAPG188E
- - + - XIAPG188R
- + - - XIAPWT
IP: α-FLAG
USP9X
*
XIAP (α-FLAG)
WCE
USP9X
XIAP (α-FLAG)
β-Actin

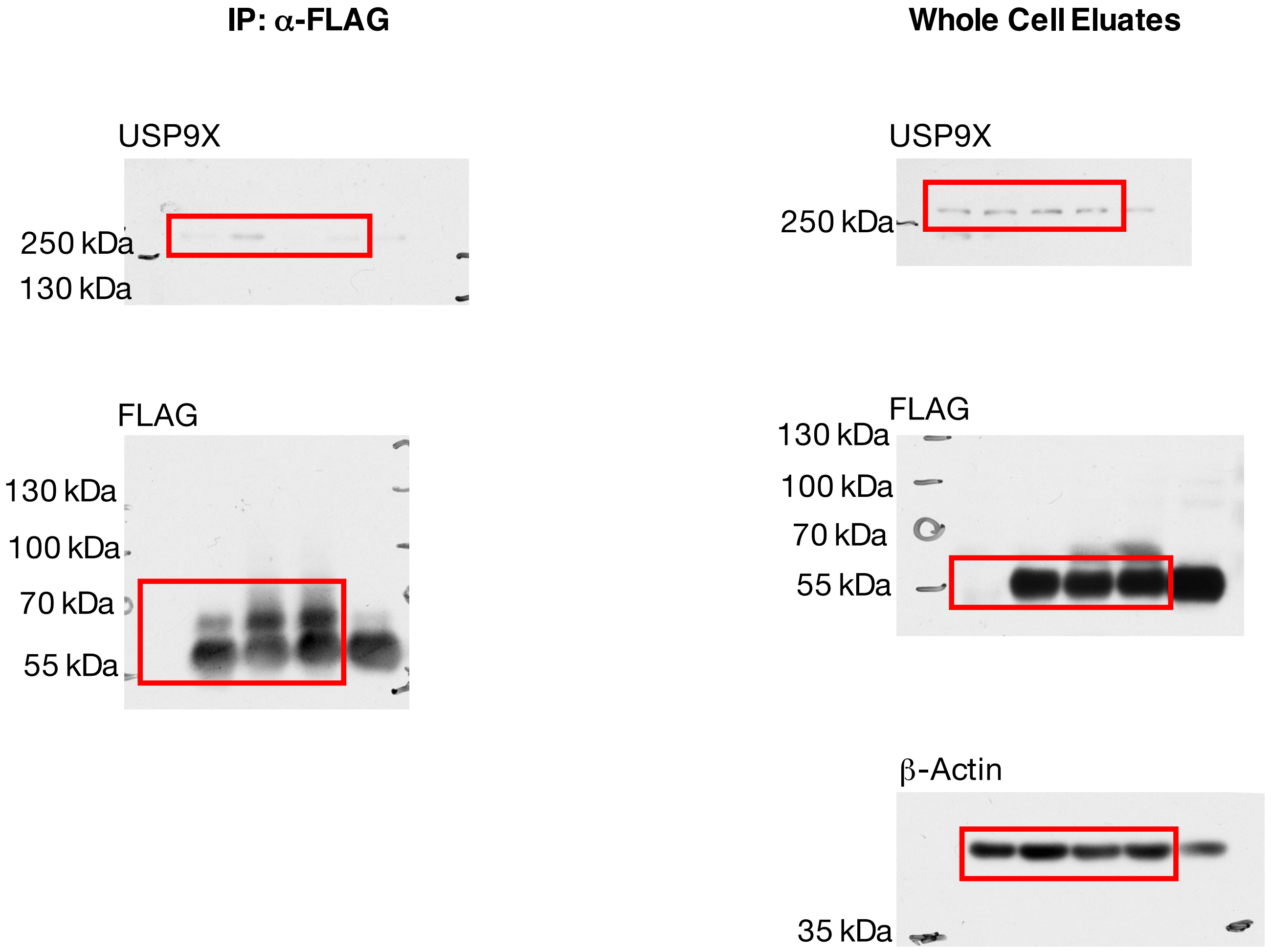

IP: α-FLAG
Whole Cell Eluates
USP9X
250 kDa
130 kDa
USP9X
250 kDa
FLAG
130 kDa
100 kDa
70 kDa
55 kDa
FLAG
130 kDa
100 kDa
70 kDa
55 kDa
β-Actin
35 kDa

Figure 1

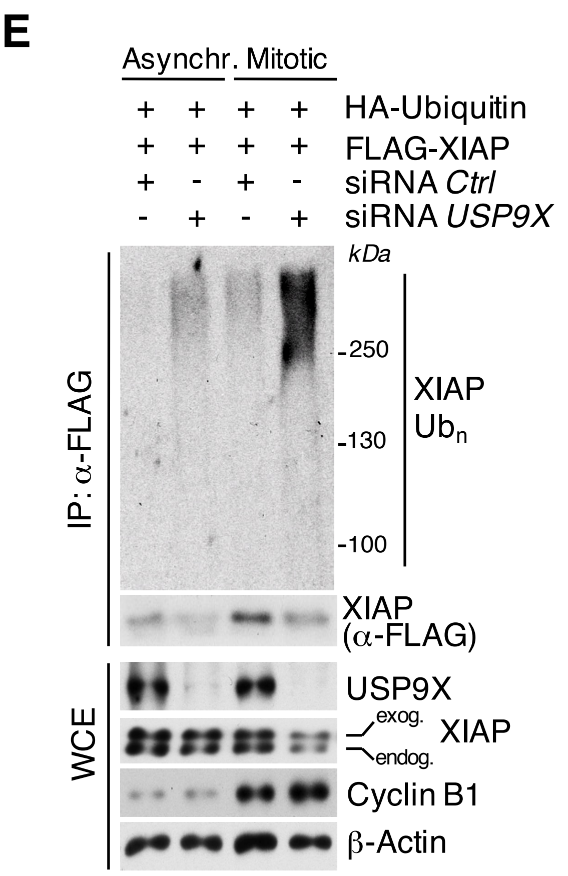
E
Asynchr.
Mitotic
HA-Ubiquitin
FLAG-XIAP
siRNA *Ctrl*
siRNA *USP9X*
kDa
250
130
100
XIAP Ub$_n$
XIAP (α-FLAG)
IP: α-FLAG
USP9X
exog.
endog.
XIAP
Cyclin B1
β-Actin
WCE

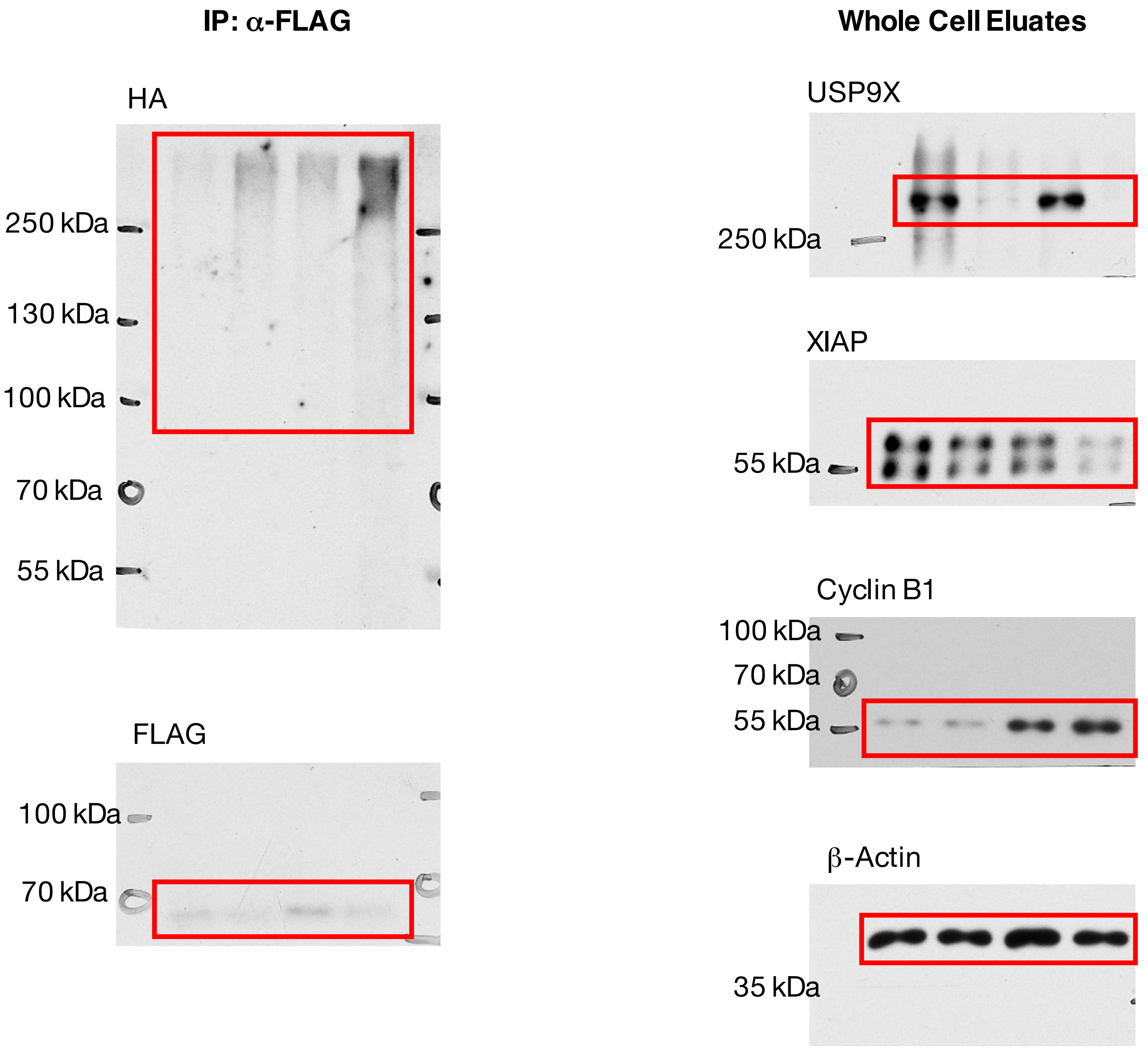
IP: α-FLAG
Whole Cell Eluates
HA
250 kDa
130 kDa
100 kDa
70 kDa
55 kDa
FLAG
100 kDa
70 kDa
USP9X
250 kDa
XIAP
55 kDa
Cyclin B1
100 kDa
70 kDa
55 kDa
β-Actin
35 kDa

Figure 1

F

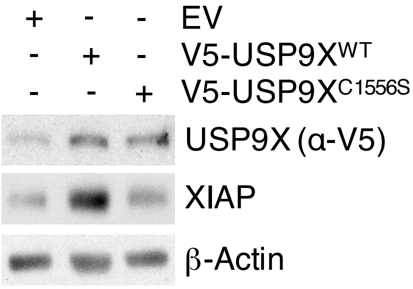

+ - - EV
- + - V5-USP9X^{WT}
- - + V5-USP9X^{C1556S}
USP9X (α-V5)
XIAP
β-Actin

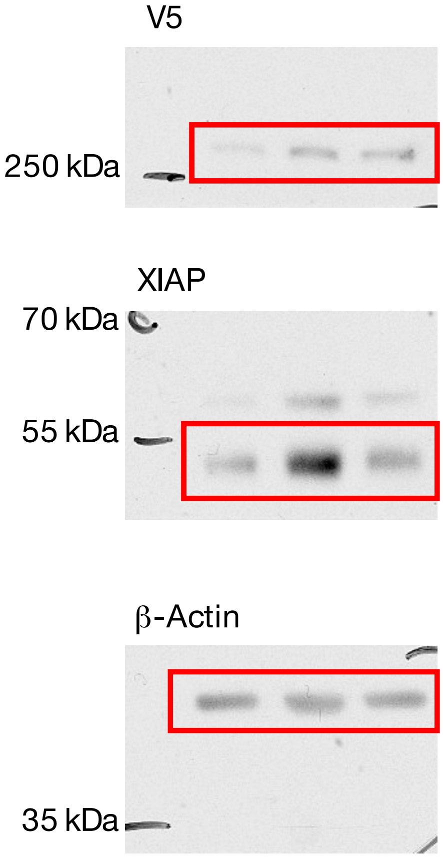

V5
250 kDa
XIAP
70 kDa
55 kDa
β-Actin
35 kDa

Figure 1

G

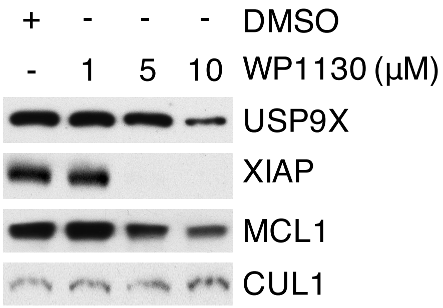

+ - - - DMSO
- 1 5 10 WP1130 (µM)
USP9X
XIAP
MCL1
CUL1

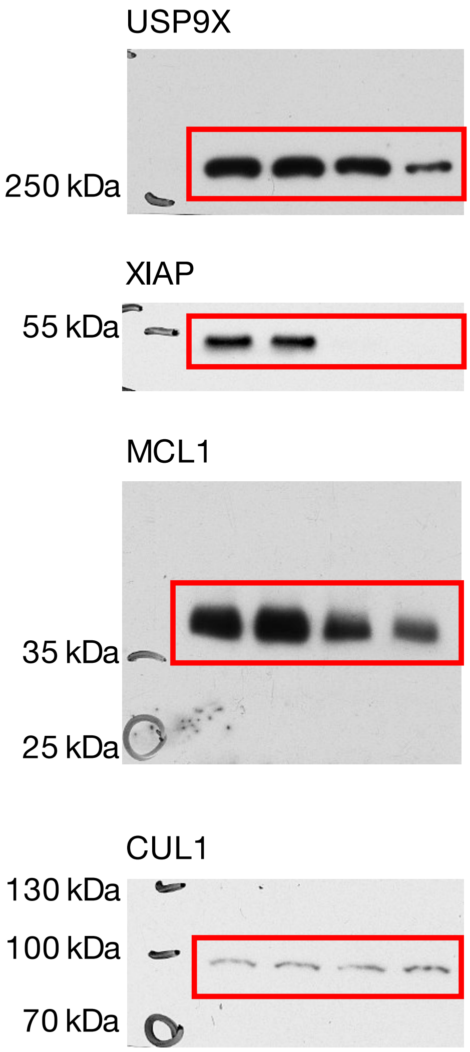

USP9X
250 kDa
XIAP
55 kDa
MCL1
35 kDa
25 kDa
CUL1
130 kDa
100 kDa
70 kDa
