# Supplementary material for: USP9X stabilizes XIAP to regulate mitotic cell death and chemoresistance in aggressive B‐cell lymphoma
Source: EMBO Mol Med. 2016 Jun 17;8(8):851–62. doi: 10.15252/emmm.201506047 (PMC4967940; doi:10.15252/emmm.201506047)
Supplement: Supplementary file 6 — Source Data for Figure 2 [file EMMM-8-851-s005.pdf]

Figure 2

A.I

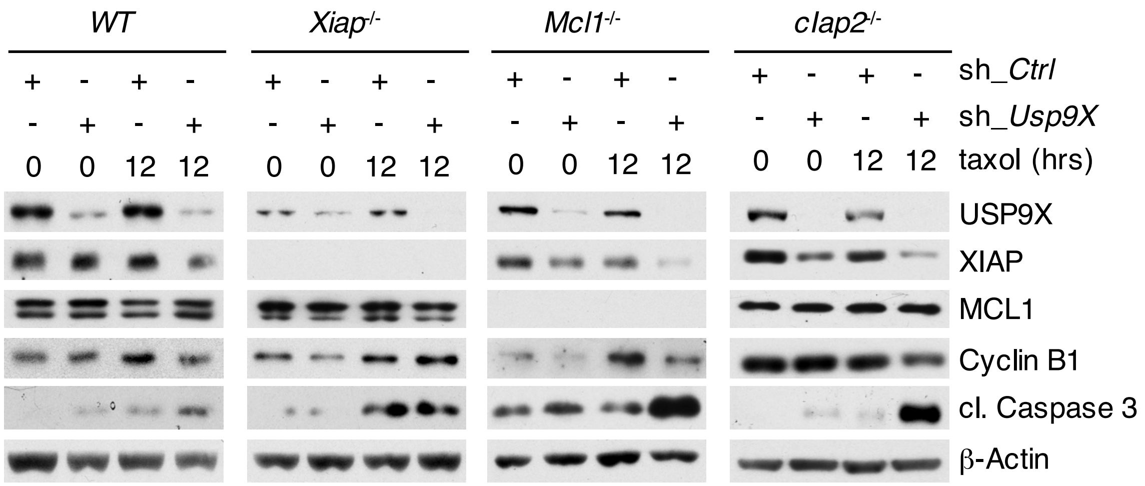

WT
Xiap-/-
Mcl1-/-
cIap2-/-
sh_Ctrl
sh_Usp9X
taxol (hrs)
+ - + -
- + - +
0 0 12 12
USP9X
XIAP
MCL1
Cyclin B1
cl. Caspase 3
β-Actin

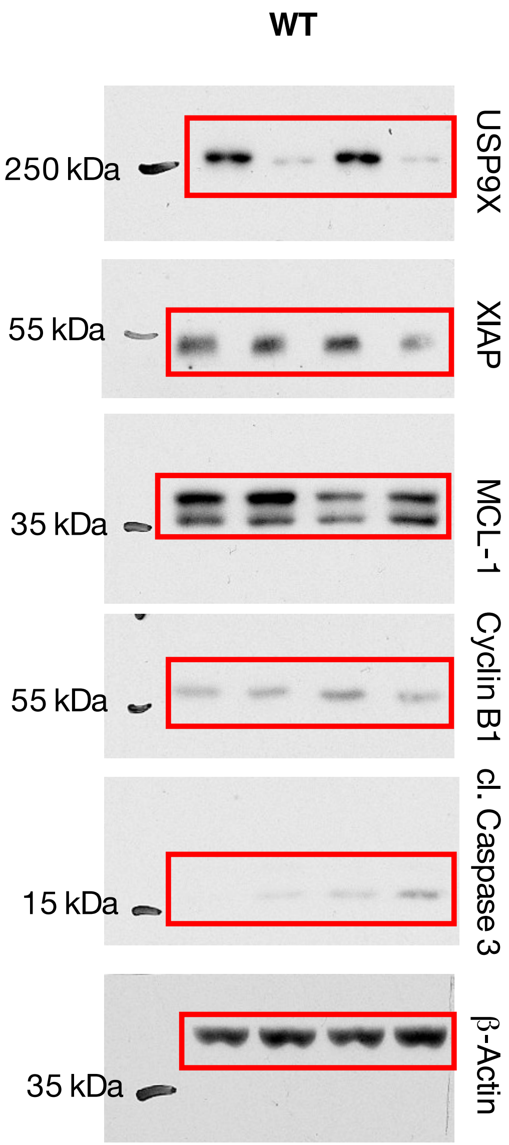

WT
250 kDa
USP9X
55 kDa
XIAP
35 kDa
MCL-1
55 kDa
Cyclin B1
15 kDa
cl. Caspase 3
35 kDa
β-Actin

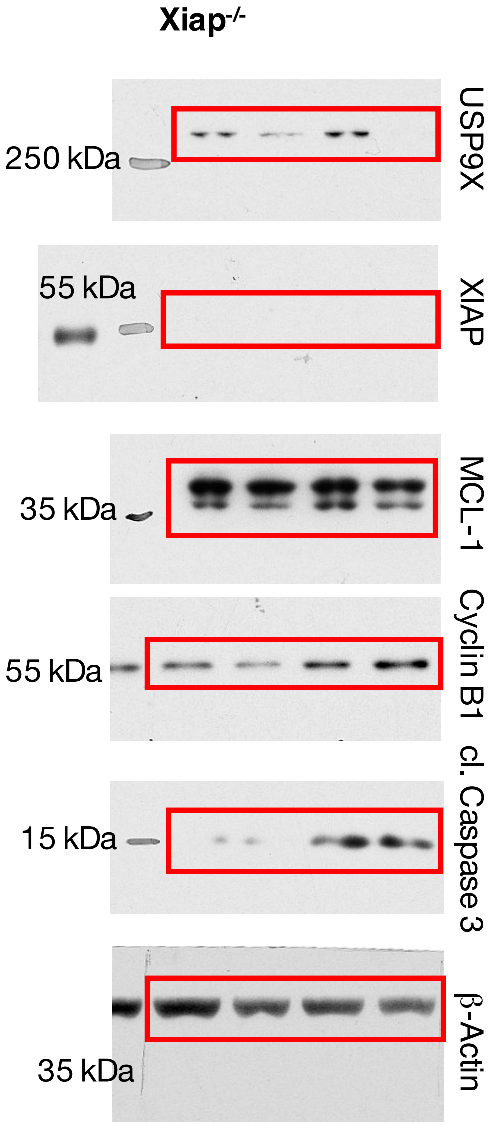

Xiap-/-
250 kDa
USP9X
55 kDa
XIAP
35 kDa
MCL-1
55 kDa
Cyclin B1
15 kDa
cl. Caspase 3
35 kDa
β-Actin

Figure 2

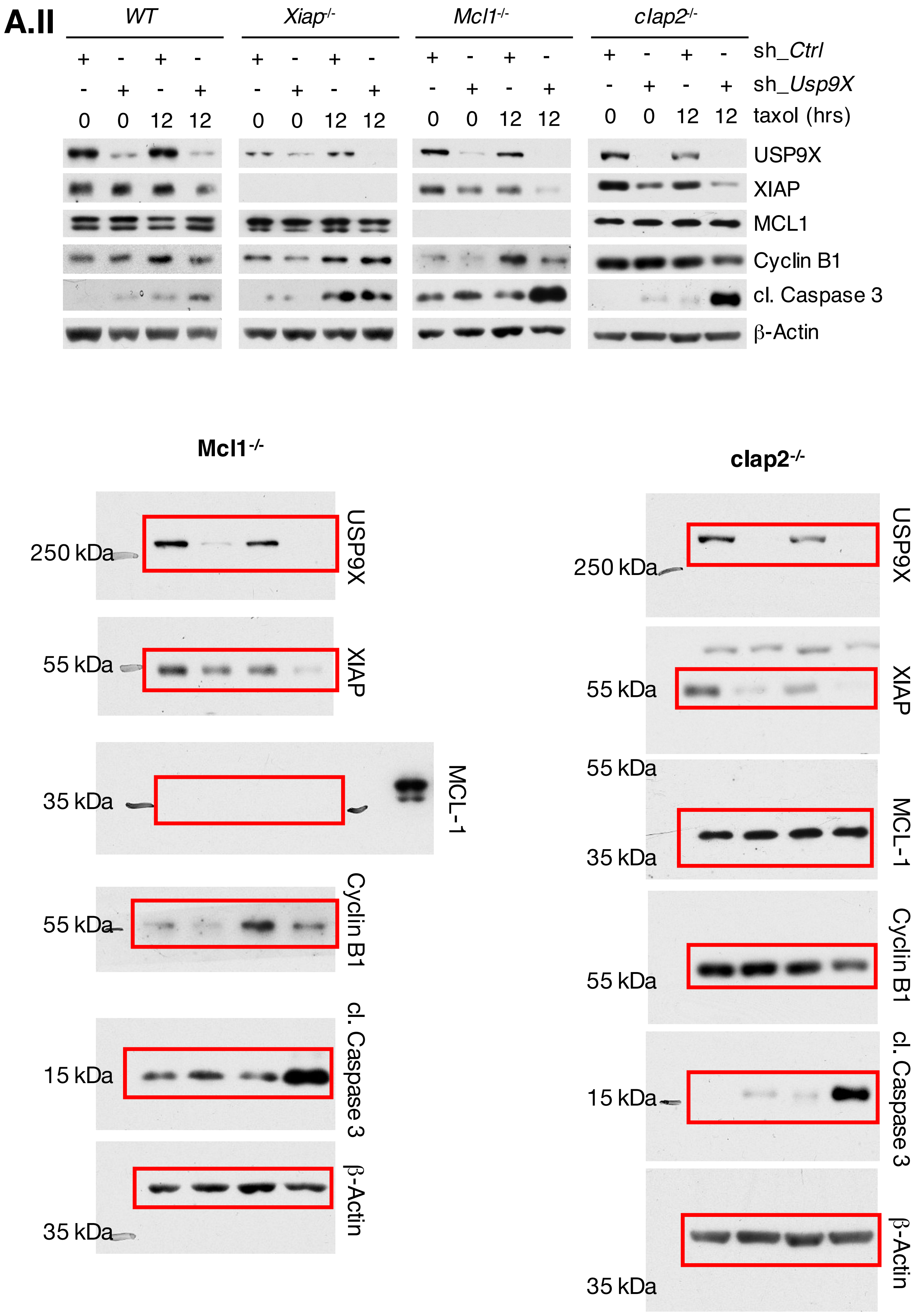
A.II
WT
Xiap-/-
Mcl1-/-
cIap2-/-
sh_Ctrl
sh_Usp9X
taxol (hrs)
+ - + -
- + - +
0 0 12 12
USP9X
XIAP
MCL1
Cyclin B1
cl. Caspase 3
β-Actin
Mcl1-/-
cIap2-/-
250 kDa
55 kDa
35 kDa
15 kDa
USP9X
XIAP
MCL-1
Cyclin B1
cl. Caspase 3
β-Actin

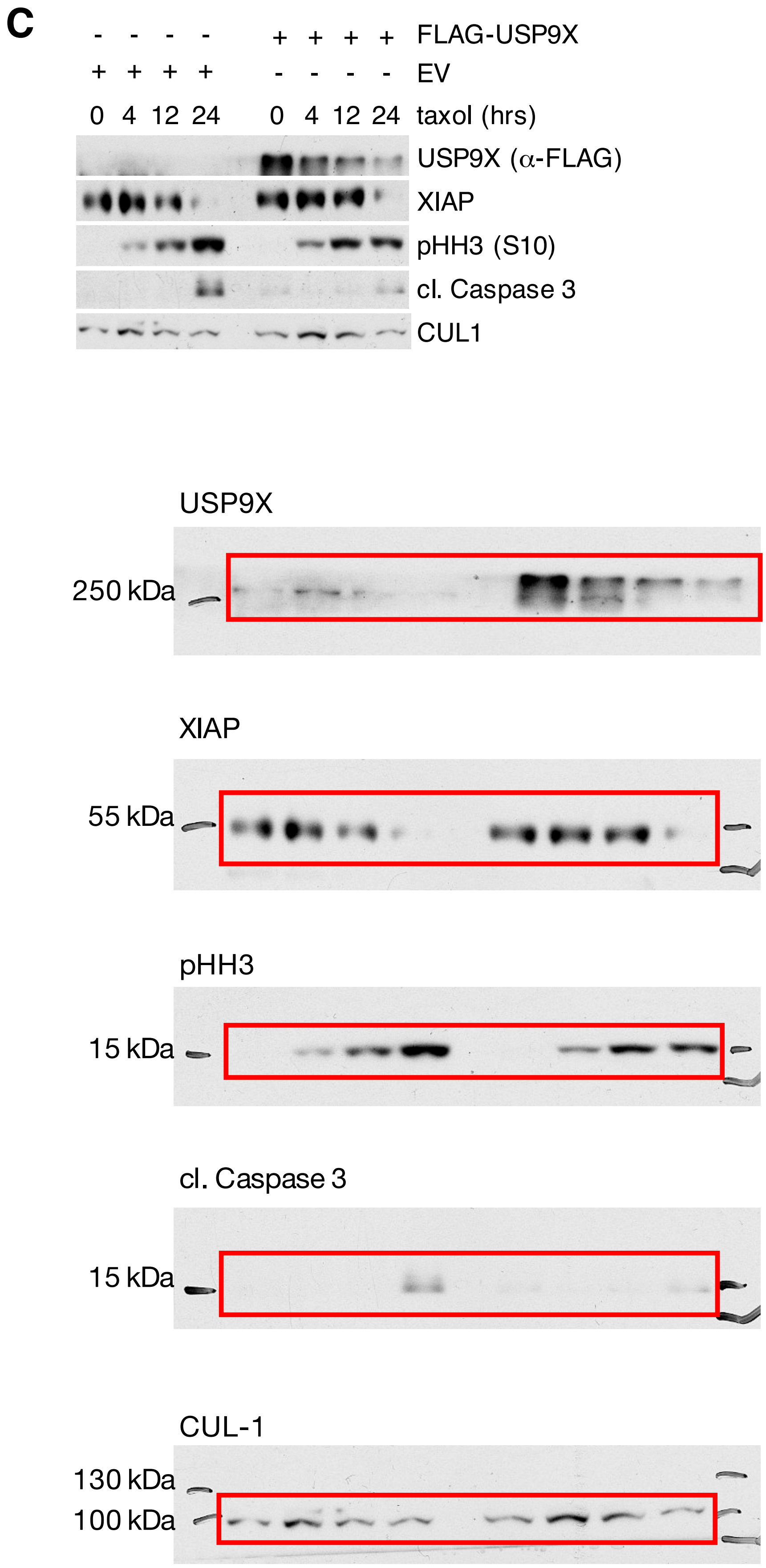
c
- - - - + + + + FLAG-USP9X
+ + + + - - - - EV
0 4 12 24 0 4 12 24 taxol (hrs)
USP9X (α-FLAG)
XIAP
pHH3 (S10)
cl. Caspase 3
CUL1
USP9X
250 kDa
XIAP
55 kDa
pHH3
15 kDa
cl. Caspase 3
15 kDa
CUL-1
130 kDa
100 kDa

Figure 2

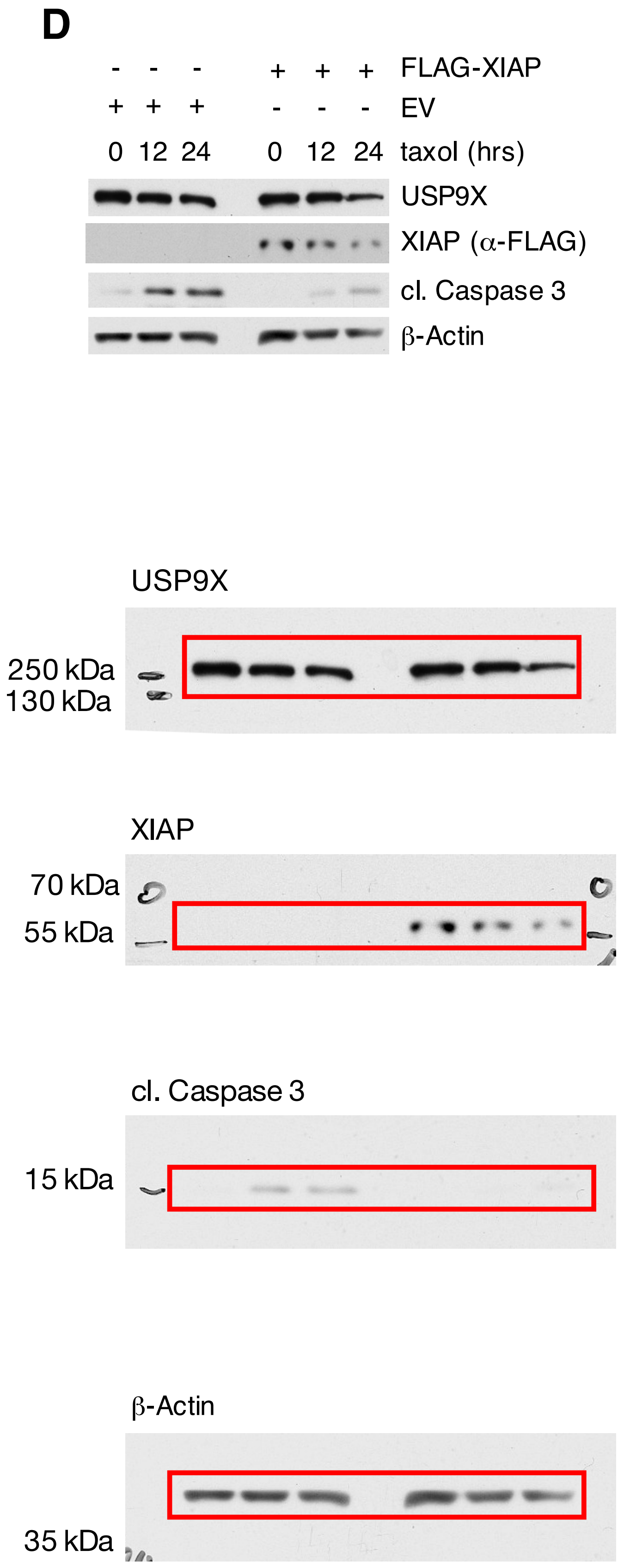
D
- - - + + + FLAG-XIAP
+ + + - - - EV
0 12 24 0 12 24 taxol (hrs)
USP9X
XIAP (α-FLAG)
cl. Caspase 3
β-Actin
USP9X
250 kDa
130 kDa
XIAP
70 kDa
55 kDa
cl. Caspase 3
15 kDa
β-Actin
35 kDa

Figure 2

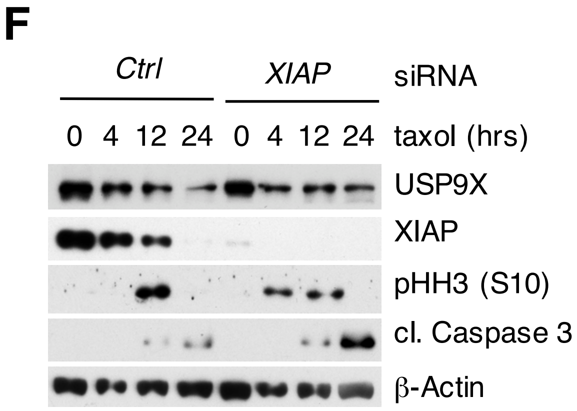
F
Ctrl
XIAP
siRNA
0 4 12 24 0 4 12 24 taxol (hrs)
USP9X
XIAP
pHH3 (S10)
cl. Caspase 3
β-Actin

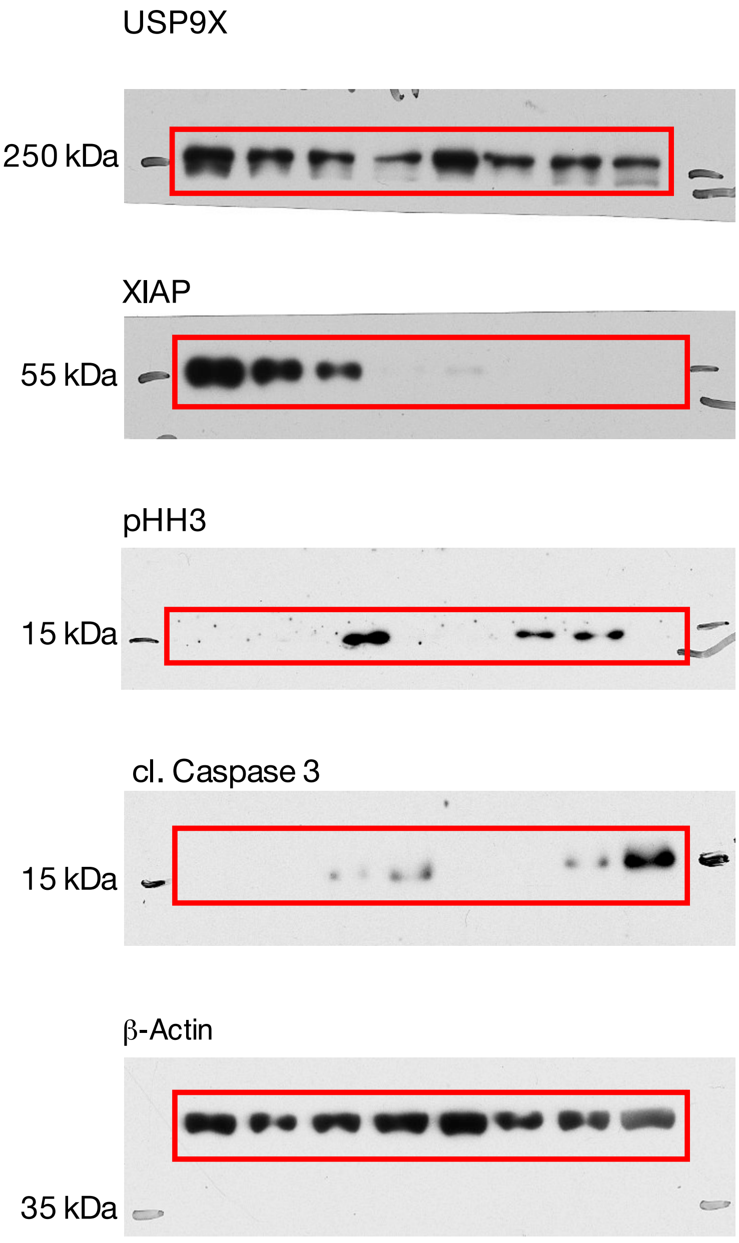
USP9X
250 kDa
XIAP
55 kDa
pHH3
15 kDa
cl. Caspase 3
15 kDa
β-Actin
35 kDa
